# microRNAs‐mediated regulation of insulin signaling in white adipose tissue during aging: Role of caloric restriction

**DOI:** 10.1111/acel.13919

**Published:** 2023-07-04

**Authors:** Patricia Corrales, Marina Martin‐Taboada, Yurena Vivas‐García, Lucia Torres, Laura Ramirez‐Jimenez, Yamila Lopez, Daniel Horrillo, Rocio Vila‐Bedmar, Eloisa Barber‐Cano, Adriana Izquierdo‐Lahuerta, Maria Peña‐Chilet, Carmen Martínez, Joaquín Dopazo, Manuel Ros, Gema Medina‐Gomez

**Affiliations:** ^1^ Department of Basic Sciences of Health, Area of Biochemistry and Molecular Biology Universidad Rey Juan Carlos, Alcorcon Madrid Spain; ^2^ Metabolism and Cell Signalling Laboratory Spanish National Cancer Research Centre Madrid Spain; ^3^ Genomics and Genetics Unit Centro de Investigación Príncipe Felipe Valencia Spain; ^4^ Platform for Computational Medicine, Fundación Progreso y Salud Sevilla Spain; ^5^ Systems and Computational Medicine Unit, Biomedical Institute of Seville (IBiS) Sevilla Spain; ^6^ Plataforma BiER, Consorcio de Investigaciones Biomédicas en Red en Enfermedades Raras (CIBERER) Sevilla Spain

**Keywords:** adipose tissue, aging, caloric restriction, insulin signaling, miRNA

## Abstract

Caloric restriction is a non‐pharmacological intervention known to ameliorate the metabolic defects associated with aging, including insulin resistance. The levels of miRNA expression may represent a predictive tool for aging‐related alterations. In order to investigate the role of miRNAs underlying insulin resistance in adipose tissue during the early stages of aging, 3‐ and 12‐month‐old male animals fed ad libitum, and 12‐month‐old male animals fed with a 20% caloric restricted diet were used. In this work we demonstrate that specific miRNAs may contribute to the impaired insulin‐stimulated glucose metabolism specifically in the subcutaneous white adipose tissue, through the regulation of target genes implicated in the insulin signaling cascade. Moreover, the expression of these miRNAs is modified by caloric restriction in middle‐aged animals, in accordance with the improvement of the metabolic state. Overall, our work demonstrates that alterations in posttranscriptional gene expression because of miRNAs dysregulation might represent an endogenous mechanism by which insulin response in the subcutaneous fat depot is already affected at middle age. Importantly, caloric restriction could prevent this modulation, demonstrating that certain miRNAs could constitute potential biomarkers of age‐related metabolic alterations.

## Graphical abstract

At middle age there is insulin resistance in subcutaneous white adipose tissue, which is improved by caloric restriction. Differentially expressed miRNAs in this tissue during aging may modulate key genes implicated in the insulin signaling pathway, leading to the progressive appearance of insulin resistance during aging. The beneficial effects of caloric restriction on insulin sensitivity and glucose uptake during aging could be mediated by the regulation of the expression of miRNAs in the subcutaneous fat depot.

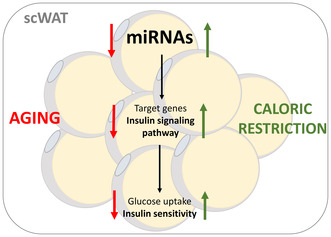

**Abbreviations**
AKT: Protein kinase B
AMPK: AMP‐Activated Protein Kinase
AT: Adipose tissue
CR: Caloric restriction
eWAT: epididymal white adipose tissue
GLUT‐4: Glucose transporter 4
Insr: Insulin receptor
IR: Insulin resistance
Irs: Insulin receptor substrate
miRNA: microRNA
mTOR: Mammalian target of rapamycin
PI3K: Phosphoinositide 3‐kinase
Pik3r1: Phosphoinositide 3‐kinase regulatory unit 1
Pten: Phosphatase and tensin homolog
Ptpn1: Protein‐tyrosine phosphatase 1B
scWAT: Subcutaneous white adipose tissue
Slc2a4: Solute carrier family 2 member 4

## 1 INTRODUCTION, RESULTS, AND DISCUSSION

Aging is a complex process correlated with the gradual loss of the physiological and metabolic function (López‐Otín et al., 2013). Thus, achieving a “healthy aging” represents a strategy for improving the organism health (Harman, 1981; Longo et al., 2015). In this context, caloric restriction (CR) without malnutrition has been proven to be the non‐pharmacological most efficient intervention to delay the deleterious effects of age‐related metabolic diseases (Colman et al., 2014; Redman et al., 2018; Ros & Carrascosa, 1866).

Lately, a few studies have suggested that CR induces changes in the microRNAs (miRNAs) expression levels (Dhahbi et al., 2013; Mercken et al., 2013; Schneider et al., 2017). miRNAs are single‐strand RNA molecules of about 22–25 bases of length involved in the regulation of posttranscriptional gene expression (Bartel, 2018). Age‐related changes in miRNAs expression have been described in a wide range of biological functions of most organs (Arner & Kulyté, 2015; Makwana et al., 2017; Mercken et al., 2013). Adipose tissue (AT) plays an important role in the development of age‐related alterations (Arner et al., 2019) and CR has been reported to impact on the AT function (Corrales et al., 2019; Miller et al., 2017). Therefore, aging and CR‐induced changes in miRNA expression within fat depots may be involved in the control of insulin signaling pathway. Here, we show the impact of long‐term CR on the miRNA regulation of insulin signaling in AT at middle age.

**FIGURE 1:**
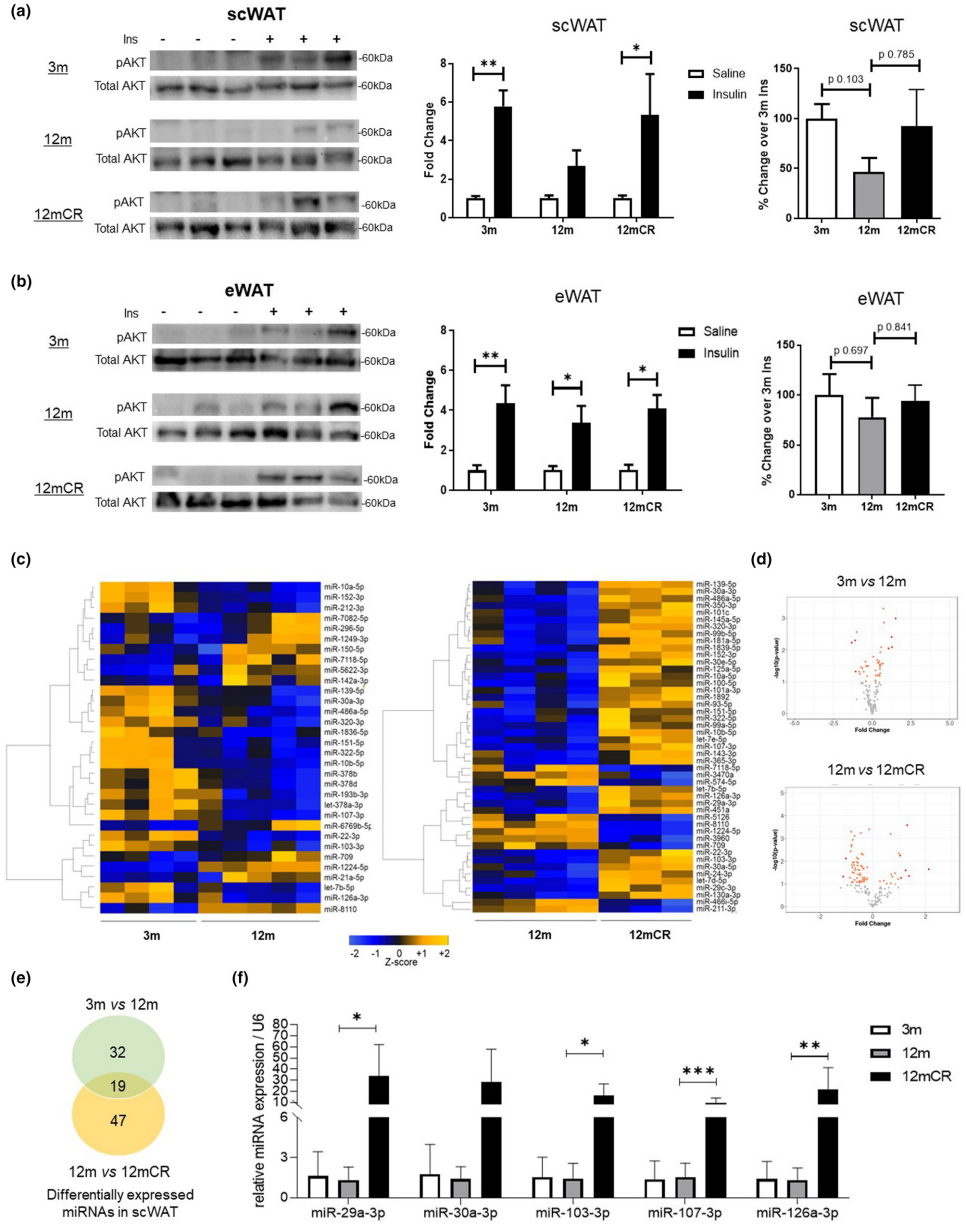
Effect of age and caloric restriction on insulin signaling in adipose tissues and miRNAs expression in scWAT. (a, b) 3 m, 12 m, and 12 mCR mice were injected with either saline (NaCl 0.9%) or insulin (10 U/kg body weight) and after 10 min, tissues were collected. AKT phosphorylation was assessed by western blotting in the subcutaneous white adipose tissue (scWAT) (a) and epidydimal white adipose tissue (eWAT) (b). The protein bands were quantified, and the fold change was calculated with respect to the control group (saline). The percentage of change over insulin‐treated 3 m mice between groups was represented and statistical differences analyzed (*p < 0.05, **p < 0.01, ***p < 0.001, insulin vs. saline; *n* = 4–6 animals/group). (c) Heatmap representing the quantitation of miRNAs expressed in the subcutaneous white adipose tissue (scWAT) of mice at 3 and 12 months of age fed ad libitum (3 m and 12 m) and at 12 months of age after a long‐term of caloric restriction (12mCR). (d) Volcano plot representing the quantification of miRNAs expressed in the subcutaneous white adipose tissue (scWAT) of mice at 3 and 12 months of age fed ad libitum (3 m and 12 m) and at 12 months of age after a long‐term of caloric restriction (12mCR). (e) Venn diagram showing interactions between differentially expressed miRNAs (logFC ≥ 1 and logFC < −1; adj. *p*‐value <0.05) in the comparisons 3 m versus 12 m and 12 m versus 12 mCR. (f) Quantification of expression of selected miRNAs by qRT‐PCR in scWAT of aged and caloric restricted mice (*p < 0.05; **p < 0.01; *n* = 3–6 animals/group).

This study was conducted using male mice at 3 and 12 months of age fed ad libitum (3 m and 12 m), defined as young and middle‐aged mice, respectively. A third group of 12‐month‐old mice were fed under a 20% of CR (12mCR). Our previous studies showed that 12‐month‐old mice had peripheral insulin resistance (IR), probably related to the alterations of AT functionality, which were importantly improved by CR (Corrales et al., 2019; Table S1). Along this line, we next studied the activation status of the insulin pathway between the experimental groups in the most insulin responsive tissues. Although the impaired subcutaneous white AT (scWAT) function with aging has been widely associated with age‐related metabolic disorders (Spinelli et al., 2020; Stout et al., 2014, 2017), the contribution of this tissue on the overall insulin sensitivity has not been fully elucidated. In this work, middle‐aged mice showed IR in the scWAT, observed by a defect in AKT activation after insulin stimulation (Figure 1a). CR, in turn, restored AKT phosphorylation and insulin sensitivity in this depot (Figure 1a). The impairment on insulin action in scWAT at 12 months of age was not observed, at least to the same extent, in epididymal WAT (eWAT) (Figure 1b), liver or gastrocnemius muscle (Figure S1A,B), suggesting that the lack of insulin responsiveness in scWAT may be contributing to the overall peripheral IR observed at this age and the amelioration of this failure by CR (Corrales et al., 2019).

miRNAs play an important role in metabolic homeostasis by regulating metabolic signaling pathways, such as the insulin pathway (Chakraborty et al., 2014; Pérez‐García et al., 2022; Rottiers & Näär, 2012; Ying et al., 2017). In order to identify potential miRNAs that could modulate the AT insulin‐signaling pathway at middle age, we performed a microarray. The miRNA expression array in scWAT revealed 32 deregulated miRNAs in 12 m versus 3 m and 47 deregulated miRNAs in 12 mCR versus 12 m (Figure 1c,d). The interactions between both comparisons revealed 19 matching miRNAs (Figure 1e, Table S2). Most of these miRNAs showed a decreased expression at middle age which was significantly prevented with CR (Table S2), suggesting that the regulation of miRNAs is a potential mechanism through which aging modulates scWAT function, as previously observed in other animal models (de Lencastre et al., 2010; Mori et al., 2012). To validate the microarray data, the levels of miRNA expression were assessed by qRT‐PCR (Figure 1f) with five selected miRNAs obtained from an independent cohort with the same age and characteristics of the animals used to assess the microarray (Table S3). These miRNAs were selected based on previous relevant literature in the field and had a differential expression across groups with a significant p‐value, confirming the differences among the experimental groups (Table S3). Specifically, expression of miR‐29a‐3p, miR‐30a‐3p, miR‐103‐3p, miR‐107‐3p, and miR‐126a‐3p was similar in scWAT in young and at middle age and increased significantly with CR, except for miR‐30a‐3p, whose expression showed a tendency toward an increase in restricted animals (Figure 1f). Of note, all the miRNAs tested were indeed differentially expressed among experimental groups in the microarrays, as expected; however, no statistically significant values could be obtained by qRT‐PCR when comparing 3 m mice with middle‐aged mice.

**FIGURE 2:**
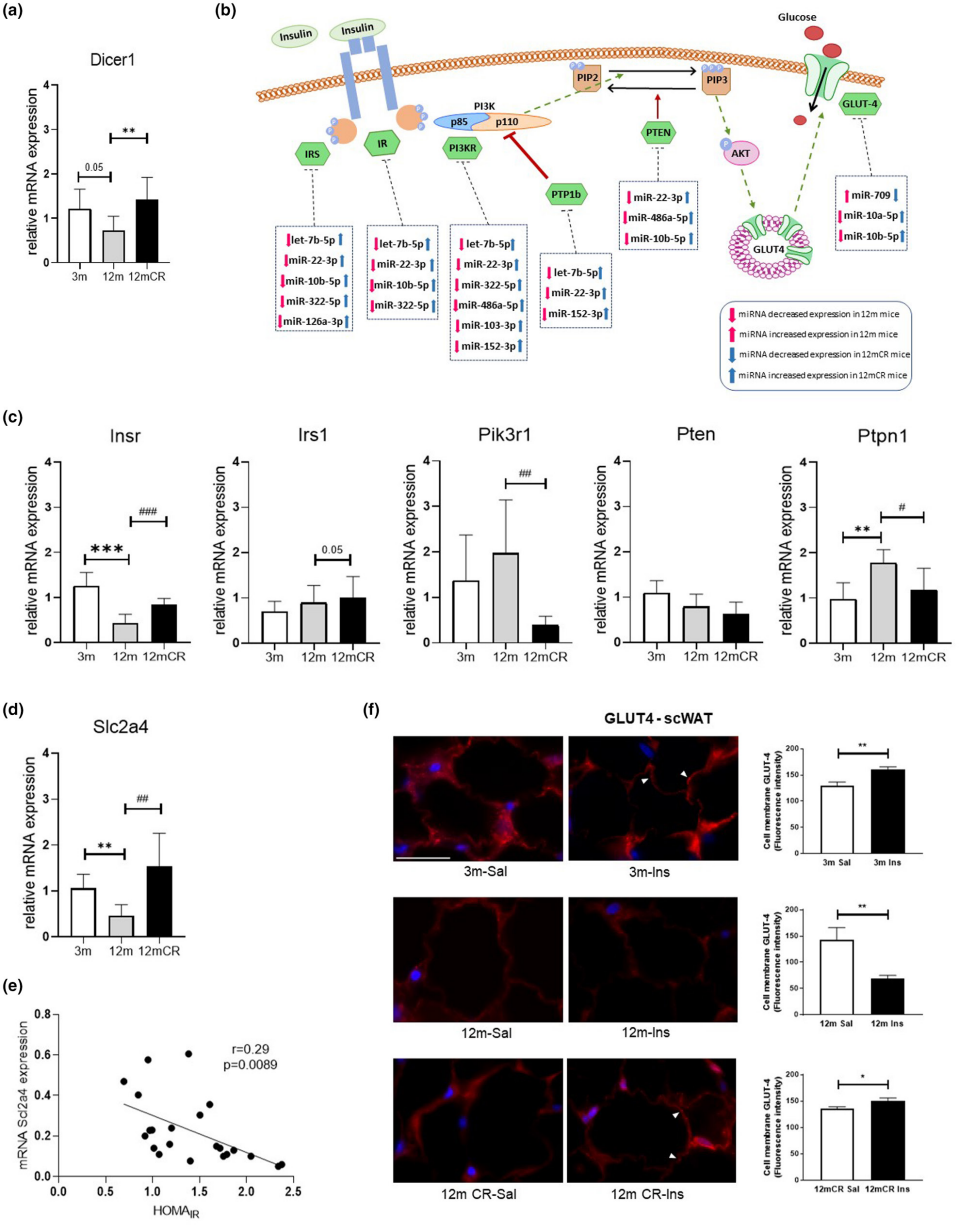
Effect of age and caloric restriction on target gene expression in subcutaneous white adipose tissue. (a) Quantification of *Dicer1* mRNA expression by qRT‐PCR in scWAT (**p < 0.01; *n* = 7–8 animals/group). (b) Schematic representation of the insulin signaling pathway showing the associations between the matching miRNAs between the 3 m versus 12 m and 12 m versus 12 mCR comparisons and the proteins encoded by the target genes, resulting from KEGG enrichment analysis. Up and down arrows show the increased or decreased expression of microRNAs with aging and caloric restriction. (c, d) Expression of mRNA levels of genes modulated by miRNAs‐*Insr, Irs1, Pik3r1, Pten, Ptpn1, and Slc2a4*‐, measured by qRT‐PCR (*p < 0.05, **p < 0.01, ***p < 0.001, 12 m versus 3 m; ^#^ *p* < 0.05, ^##^ *p* < 0.01, ^###^ *p* < 0.001, 12 mCR versus 12 m; *n* = 7–8 animals/group). (e) *Slc2a4* mRNA expression correlation with physiological HOMA_IR_. (f) Representative images of the cellular localization and trafficking of GLUT4 glucose transporter in insulin‐responsive scWAT, measured by immunofluorescence (magnification 1000×, scale bar = 20 μm). The arrowheads point to membrane‐localized GLUT4. Graphs show the quantification of the fluorescence intensity of the cell membrane‐localized GLUT‐4 (*p < 0.05, **p < 0.01, insulin vs. saline).

miRNA expression in scWAT at middle age could decline because of aging‐associated alterations in the miRNA‐processing enzyme Dicer. Dicer is responsible of cleaving the precursor miRNAs in the cytoplasm to give mature miRNAs (Jinek & Doudna, 2009). By this reason, we analyzed the expression of *Dicer1*. The data showed a downregulation of *Dicer1* expression at middle age, which was reversed by CR in scWAT (Figure 2a). A decline in *Dicer1* and miRNA processing with age have also been observed in cultured human preadipocytes and in other animal models in which this mechanism leads to changes in miRNA expression and affect the development of metabolic diseases (Mori et al., 2012, 2014; Rogers et al., 2012).

Considering that miRNAs may target from a small to a very large number or mRNA transcripts, deregulation of a set of miRNAs could have a potential impact on several biological pathways. To establish a possible biological implication of the deregulated miRNAs from the scWAT we performed in silico analyses (Supporting information). First, we performed this analysis in the comparisons 12 m versus 3 m and 12 mCR versus 12 m. These data revealed the glucose input regulation, the possible phosphorylation status, and activity of proteins implicated in the insulin pathway (*p*‐value <0.05), including MAPK (mitogen‐activated protein kinase), mTOR (mammalian target of rapamycin), thyroid hormones, PI3K/AKT (phosphoinositide 3‐kinase/protein kinase B), and AMPK (AMP‐activated protein kinase) signaling, among others (Tables S4–S9). Moreover, with the 19 matching miRNAs, we also performed in silico analyses and database searches to determine which target genes might be biologically relevant in the scWAT context (*p*‐value < 0.05 and a great number of miRNAs differentially expressed) and revealed pathways and target genes implicated in several biological processes and molecular functions related to insulin sensitivity (Table S10). The most frequent target genes regulated by the miRNAs in the scWAT (Figure 2b and Figure S2A) were key genes in the insulin pathway: *Pik3r1* (phosphoinositide‐3‐kinase regulatory unit 1), *Irs1* and *4* (insulin receptor substrate 1 and 4), *Insr* (insulin receptor), *Slc2a4* (solute carrier family 2 member 4, which encodes for the glucose transporter 4, Glut‐4), *Pten* (phosphatase and tensin homolog) and *Ptpn1* (protein‐tyrosine phosphatase 1B).

As the deregulation of these target genes may play an important role in the impaired insulin sensitivity at middle age, we studied the expression of these genes in scWAT of a new cohort of mice by qRT‐PCR (Figure 2c). Results revealed that the expression of *Insr* decreased at 12 m and was restored with CR. A tendency toward an increase in *Irs1* expression by CR was also observed. No differences were found in the expression of *Pten* among groups. Moreover, *Ptpn1*, a negative regulator of the insulin signaling pathway, was significantly increased at middle age, and decreased with CR. Although the expression levels of *Pik3r1* did not differ in middle‐aged animals, a significant decrease in the expression of the regulatory subunit was observed with CR. Importantly, *Slc2a4* expression decreased in this depot at middle age and was restored in CR mice (Figure 2d).

To establish a direct cause–effect relationship between IR and the modulation of key genes by miRNAs in scWAT, we performed a correlation analysis between the HOMA_IR_ and *Slc2a4* mRNA expression. The mRNA levels of *Slc2a4* negatively correlated with the HOMA_IR_ (Figure 2e and Figure S2B), accordingly with the IR observed in this depot. Of note, the mRNA levels of *Slc2a4* increased with long‐term CR (Figure 2d), suggesting an association between the recovery of miRNAs levels and the expression of key genes of the insulin pathway. Other correlation analyses showed a positive correlation between *Insr* and *Slc2a4* mRNA levels, and between *Dicer1* and *Slc2a4* mRNA levels (Figure S2C,D). The expected decrease in glucose uptake in scWAT at middle age was also confirmed by a decreased translocation of the transporter Glut‐4 to the adipocyte membrane, observed by immunofluorescence, demonstrating the failure of the insulin response, whereas the presence of this transporter in the membrane was recovered by CR (Figure 2f).

In agreement with the fact that no significant differences were yet observed in insulin signaling in eWAT, the microarray only revealed 15 deregulated miRNAs in eWAT in 12 m versus 3 m, and 7 deregulated in 12 mCR versus 12 m (Figure S3A,B). The expression of *Dicer1* did not change (Figure S3C), suggesting a much slighter alteration in miRNA processing, in contrast with other studies (Mori et al., 2012). Importantly, no differences in the expression of the target genes involved in the insulin signaling pathway were observed in eWAT (Figure S3D,E), nor in the GLUT‐4 localization at 12 months of age (Figure S3F). All together, these results suggest that both depots, scWAT and eWAT, have a different miRNA expression profile in male mice during aging. Nevertheless, considering the sexual dimorphism along the aging process, further studies would be needed in females to completely understand the miRNA expression profile in the different adipose depots during aging.

In summary, our work demonstrates that a set of deregulated miRNAs could modulate insulin sensitivity in the scWAT at middle age through the regulation of the expression of genes involved in the insulin signaling pathway, and that these miRNAs‐based mechanisms are rescued by long‐term CR to ameliorate the early effects of aging. Interventions that preserve miRNAs expression and processing may provide a new approach for preventing AT alterations associated with aging and other age‐related diseases such as IR.

## AUTHOR CONTRIBUTIONS

P.C., M.M.T., and G.M.G. conceived the study, researched data, and wrote the manuscript. L.R.J. and E.B.C. performed the microarray assays and M.P.C. and J.D. performed data analyses. All the co‐authors read and approved the manuscript (Y.V.G., L.T., L.R.J, Y.L., D.H., R.V.B., A.I.L., E.B.C., M.P.C., C.M., J.D., and M.R.). M.R. revised the manuscript. P.C., M.M.T., and G.M.G. are the guarantors of this work. The authors declare that there is no duality of interest associated with this manuscript.

## FUNDING INFORMATION

This work was supported by grants from Community of Madrid (Found action by the Community of Madrid in the framework of the Multiannual Agreement with the Rey Juan Carlos University in line of action 1, “Encouragement of Young PhD investigation”, A‐485‐EPIGENIDAD to P.C. and S2017/BMD‐3684 and P2022/BMD‐7227 to G.M.G.) and the Spanish Ministry of Economy and Competitiveness (BFU2013‐47384‐R, BFU2016‐78951‐R, and BFU2017‐90578‐REDT) and Spanish Ministry of Science and Innovation (PID2020‐116875RB‐I00) to G.M.G and “Ayuda a la Investigación Ignacio Hernando de Larramendi 2014” from Mapfre Foundation to G.M.G.

## CONFLICT OF INTEREST STATEMENT

The authors report no conflict of interest financial or otherwise.

### OPEN RESEARCH BADGES

This article has earned Open Data and Preregistered Research Designs badges. Data and the preregistered design and analysis plan are available at [https://doi.org/10.21950/DQZQD2, GSE217353 from ncbi.nlm.nih.gov/geo and ncbi.nlm.nih.gov/geo, GSE217353; https://doi.org/10.21950/DQZQD2 from www.consorciomadrono.es, registration for the Universidad Rey Juan Carlos].

## Supporting information


Appendix S1.

## Data Availability

The data that support the findings of this study are openly available in e‐cienciaDatos at [https://doi.org/10.21950/DQZQD2], Repositorio de Datos URJC and in GSE217353 from ncbi.nlm.nih.gov/geo.
