# Supplementary material for: microRNAs‐mediated regulation of insulin signaling in white adipose tissue during aging: Role of caloric restriction
Source: Aging Cell. 2023 Jul 4;22(11):e13919. doi: 10.1111/acel.13919 (PMC10652342; doi:10.1111/acel.13919)

**microRNAs-mediated regulation of insulin signaling in white adipose tissue during aging: role of caloric restriction**

Corrales Patricia^1*^, Martin-Taboada Marina^1*^, Vivas-García Yurena^1,2^, Torres Lucia^1^, Ramirez-Jimenez Laura^2^, Lopez Yamila^1,^ Horrillo Daniel^1^, Vila-Bedmar Rocio^1^, Barber-Cano Eloisa^2^, Izquierdo-Lahuerta Adriana^1^, Peña Maria-Chilet^4,5,6^ Martínez Carmen^1^, Dopazo Joaquín^4,5,6^, Ros Manuel^1^, Medina-Gomez Gema^1^

**Supplementary Material**

- **Experimental procedures**
- **Tables S1-S10**
- **Figure S1**
- **Figure S2**
- **Figure S3e**

**Experimental procedures**

**Animals and diets**

All animal procedures conformed to European Union laws and guidelines for animal care, and experimental procedures by the ethics committee of the Universidad Rey Juan Carlos (Spain) were approved. 3 and 12-month-old male 129S2/SvPasCrl mice (Charles River Laboratories) were used for all studies and were housed individually in climate-controlled quarters (21ºC) with a 12-hour light/dark cycle. Food (13% calories derived from fat; Research diet#2014C, Harlan Laboratories) and water were available *ad libitum* unless otherwise stated. In some experiments, mice were randomly assigned to undergo 20% CR from 3 months of age until 12 months of age, as described earlier (Sierra Rojas et al. 2016; Speakman & Mitchell 2011). The restricted food was administrated at 19 h just before the dark period starts. Animals were sacrificed by cervical dislocation and selected tissues were kept for analysis.

**Insulin stimulation**

A cohort of 4-8 male mice were fasted for 16 h and injected intraperitoneally with human insulin (Actrapid, 10 U/kg body weight: Novo Nordisk) or saline (NaCl 0.9%). White adipose tissue (subcutaneous and visceral -epidydimal-), muscle and liver samples were collected ten minutes after injection.

**Western Blotting Analysis**

Protein lysates from mouse tissues were electrophoresed in 8% SDS PAGE gels and probed with the following antibodies: phosphorylated-Ser473-Akt (Cell Signaling Technology #9271); total Akt (Cell Signaling Technology #4685). Chemiluminiscence images were generated by the ChemiDocTM System (BioRad Laboratories) and protein bands density was measured using the ImageLab 6.0 software (BioRad Laboratories). The amount of protein in control conditions was assigned with a relative value of 1.

**Immunofluorescence**

Five µm-thick sections from paraffin-embedded adipose tissue samples were rehydrated, blocked and then incubated with primary antibody GLUT-4 (Merck #07-1404) overnight at 4º C. Afterwards, slides were incubated with the secondary antibody AlexaFluor anti-rabbit IgG (JacksonImmunoResearch) for 1 h, nuclei were stained with DAPI and were directly observed in a fluorescence microscope (AxioPlan2, Zeiss). The fluorescence intensity of the positive dots in the adipocytes cell membrane labelling GLUT-4 were quantified using ImageJ.

**miRNA microarray**

In order to obtain miRNA expression, Agilent microarray (Agilent Technologies) and miRNA Complete Labeling and Hyb kit (Agilent Technologies) were used according to the miRNA Microarray System with miRNA Complete Labeling and Hyb Kit Protocol V3.1.1. First, total RNA and miRNA fraction was isolated by using TaqMan® Advanced miRNA Assays (Applied Biosystems) following the manufacturer’s protocol. RNA integrity determined by RIN (RNA integrity number) value was assessed with the Agilent 2100 Bioanalyzer using the RNA 6000 nano Bioanalyzer (Agilent Technologies). RIN value lower than 8 was considered as an exclusion criteria for the samples. After, the RNA was labeled with Cyanine 3-pCp and hybridized on the SurePrint Mouse miRNA Microarrays 8x60K microarray (AMADID number 070155) for 20 h at 55° C, containing a total of 2164 control and 62976 mouse-specific probes. Subsequently, the Agilent G2565C Microarray Scanner was used for scanning the samples. After that, data were extracted with Feature Extraction Software 11.5.11 following the protocol developed by Agilent in Agilent miR_1105_Oct12 in conjunction with the miRNA_QCMT_Oct12 metrics and using 070155_D_F_20141006 as Grid.

**Array data processing and analysis**

Data quality controls were implemented in R/Bioconductor package AgiMicroRna (Agilent). All data were normalised by the RMA (Robust Multi.Array Average) method. After filtering the data for uniformly low expression and low variance across sample, in order to determine the differences in expression pattern between groups, we carried out differential expression analyses performing a t-test by permutation testing, using genefilter R/Bioconductor package, p-values were adjusted for multiple comparisons by Benjamini & Hochberg False Discovery Rate (FDR). Those miRNAs with an adjusted p-value < 0.05 were considered statistically significant.

**Pathway enrichment analysis and candidate gene searching**

Pathway enrichment analysis was used to gain insight into global molecular networks and canonical pathways related to differentially expressed miRNAs (<http://diana.imis.athena-innovation.gr/DianaTools/index.php?r=mirpath/index>). The software performs a pathway enrichment analysis of multiple miRNA target genes comparing each set of miRNA targets as defined by the Kyoto Encyclopedia of Genes and Genomes (KEGG, <https://www.genome.jp/kegg.org>). Those analysis showing a p-value < 0.05 were considered significantly enriched between classes under comparison. Additionally, we also evaluated the functional relevance of differentially expressed miRNAs performing multidimensional enrichment analysis with R packages mdgsea and enrichr, thus uses Reactome, Gene Ontology and TRANSFAC-JASPAR databases (<https://reactome.org>, <http://geneontology.org>, <https://jaspar.genereg.net/>, respectively).

**Screening of differentially expressed miRNAs and its targets**

The 19 miRNAs that were deregulated both in 12m *vs* 3m and 12mCR *vs* 12m comparisons were analyzed using DIANA tools to identify its target KEGG pathways and genes, according to mirTarbase and microT-CDS. We selected those target pathways with enrichment adjusted p-value < 0.05 and that were enriched in 17 or more of the miRNAs evaluated.

**miRNA microarray validation by qRT-PCR**

Quantitative RT-PCR of relevant miRNAs was performed in a new cohort of animals. For selected miRNAs cDNA was synthesized by using TaqMan® Advanced miRNA Assays (Applied Biosystems) following the manufacturer’s protocol. RNA integrity determined by RIN (RNA integrity number) value was assessed with the Agilent 2100 Bioanalyzer using the RNA 6000 nano Bioanalyzer (Agilent Technologies). Specific TaqMan microRNA Assays (Applied Biosystems) probes were used for qRT-PCR by using TaqMan® Fast Advanced Master Mix (Applied Biosystems) with the standard protocol.

For data normalization, reference small RNA U6 was used. In this case, cDNA was synthesized by using TaqMan® Small RNA Assays (Applied Biosystems) following the manufacturer’s protocol. qRT-PCR for U6 was performed using TaqMan® Universal PCR Master Mix, No AmpErase® UNG (Applied Biosystems) with the standard protocol.

This study was managed using LightCycler 480 (Roche). Relative expresion was calculated by using the comparative Fit Points method and obtaining 2^-ΔΔCt^ in the LightCycler® 480 SW v1.5 (Roche). Data analyses were performed via GraphPad Prism v6.00 and Mann-Whitney’s non-parametric test was used to compare experimental groups (p-value threshold of 0.05).

**RNA Isolation and gene expression**

Gene expression of candidate genes was evaluated in order to validate results and to assess whether changes in miRNA patterns were indeed affecting target’s mRNA quantification.

Total RNA from scWAT and eWAT was isolated using miRNeasy Mini Kit (Qiagen) with homegenizator QIAshredder (Qiagen) following the manufacturer’s protocol. RNA concentration was measured using a NanoDrop ND 1000 (NanoDrop Technologies).

The input value of the gene expression of interest was normalized against β2-microglobulin, 36b4 or 18S as internal controls. All gene expression data were managed using 7500 Real-Time PCR System (Applied Biosystems). The method employed to calculate the relative index of gene expression was 2^-ΔΔCt^.

Gene expression was assessed using specific primers (SYBR Green assays or TaqMan probes) as follow:

Primers for SYBR Green assays:

| **Gene** | **Forward** | **Reverse** |
| --- | --- | --- |
| **Β2mg** | ACTGATACATACGCCTGCAGAGTT | TCACATGTCTCGATCCCAGTAGA |
| **36b4** | CTCTCGCTTTCTGGAGGGTG | ACGCGCTTGTACCCATTGAT |
| **Dicer** | CACACGCCTCCTACCACTACAACAC | CCGTGGGTCTTCATAAAGGT |
| **InsR** | AGGCTCCCGTCTCTTCTTCAA | GACATCCCCACATTCCTCGTT |
| **Irs1** | TCCAGAAGCAGCCAGAGGA | AGGATTTGCTGAGGTCATTTAGGT |
| **Pi3kr1** | GGAATTCATGGCAGGAGCCGAGGGCTTC | CGTCGACGGGCGTGCTGCAGACGGTGGGC |
| **Pten** | CAAATATTATTGCTATGGGATTTCCTGC | GCTGTGGTGGGTTATGGTCTTC |
| **Ptp1b** | AAGACCCATCTTCCGTGGAC | ACAGACGCCTGAGCACTTTG |

TaqMan probes:

| **Gene** | **Forward** | **Reverse** | **Probe** |
| --- | --- | --- | --- |
| **18S** | CGGCTACCACATCCAAGGAA | GTCGGAATTACCGCGGCT | GAGGGCAAGTCTGGTGCCAG |
| **Glut4** | ACTCATTCTTGGACGGTTCCTC | CACCCCGAAGATGAGTGGG | TGGCGCCTACTCAGGGCTAACATCA |

**Statistical analysis for miRNA and gene expression**

All data are presented as mean ± SEM. Statistical analysis was carried out with GraphPad Prism Software (San Diego, CA, USA). Results were analyzed with a Shapiro-Wilk normality test. Two experimental groups were compared using the Mann-Whitney’s non-parametric test, and three or more groups were compared by a Kruskal-Wallis test. A p value < 0.05 was considered significant.

**Methods references**

Sierra Rojas JX, García-San Frutos M, Horrillo D, Lauzurica N, Oliveros E, Carrascosa JM, Fernández-Agulló T & Ros M (2016) Differential Development of Inflammation and Insulin Resistance in Different Adipose Tissue Depots Along Aging in Wistar Rats: Effects of Caloric Restriction. J Gerontol A Biol Sci Med Sci 71, 310–22.

Speakman JR & Mitchell SE (2011) Caloric restriction. Mol Aspects Med 32, 159–221.

**Table S1. Relevant metabolic data from the experimental groups.**

|  | **3m** | **12m** | **12mCR** |
| --- | --- | --- | --- |
| **Body weight (g)** | 22.36 ± 0.42 | 34.10 ± 0.91*** | 27.25 ± 0.59 ### |
| **scWAT (g)** | 0.14 ± 0.01 | 0.33 ± 0.007 ** | 0.22 ± 0.02 # |
| **Fasting Glucose (mg/dL)** | 98.25 ± 5.40 | 100.80 ± 4.07 | 120.4 ± 9.66 # |
| **Fasting Insulin (µg/L)** | 0.22 ± 0.11 | 0.61 ± 0.06 * | 0.15 ± 0.05 # |
| **HOMA_IR_** | 1.51 ± 0.34 | 4.38 ± 0.15 ** | 1.29 ± 0.20 ### |

**Table S2. Differential expression of 19 matching miRNAs between comparisons in 3m *vs* 12m and 12m *vs* 12mCR.**

| **Downregulated miRNAs in 12m and upregulated in 12mCR** | | | | |
| --- | --- | --- | --- | --- |
| **miRNAS** | **3m *vs* 12m** | | **12m *vs* 12mCR** | |
|  | **Mean difference** | **p value** | **Mean difference** | **p value** |
| let-7b-5p | 0.5895 | 0.0273 | -0.3675 | 0.0218 |
| miR-103-3p | 0.32415 | 0.0203 | -0.8394 | 0.0037 |
| miR-107-3p | 0.7237 | 0.0005 | -0.7575 | 0.0012 |
| miR-10a-5p | 0.4801 | 0.0285 | -0.5112 | 0.0243 |
| miR-10b-5p | 0.5052 | 0.0308 | -0.6883 | 0.0008 |
| miR-126a-3p | 0.76105 | 0.0014 | -0.8043 | 0.0023 |
| miR-139-5p | 0.35275 | 0.0249 | -0.5511 | 0.0079 |
| miR-151-5p | 0.51055 | 0.0339 | -0.4811 | 0.0419 |
| miR-152-3p | 0.5794 | 0.0082 | -0.8147 | 0.0005 |
| miR-1839-5p | 0.06835 | 0.0163 | -0.1354 | 0.0004 |
| miR-22-3p | 0.5256 | 0.0117 | -0.7877 | 0.0148 |
| miR-30a-3p | 0.44255 | 0.0224 | -0.5771 | 0.0155 |
| miR-320-3p | 0.21305 | 0.0440 | -0.4233 | 0.0047 |
| miR-322-5p | 0.6937 | 0.0267 | -0.7142 | 0.0331 |
| miR-486a-5p | 0.49935 | 0.0399 | -0.5941 | 0.0108 |
| **Upregulated miRNAs in 12m and downregulated in 12mCR** | | | | |
| **miRNAS** | **3m *vs* 12m** | | **12m *vs* 12mCR** | |
|  | **Mean difference** | **p value** | **Mean difference** | **p value** |
| miR-1224-5p | -1.3188 | 0.0058 | 1.309 | 0.0003 |
| miR-709 | -0.54705 | 0.0482 | 0.8506 | 0.0336 |
| miR-7118-5p | -0.9985 | 0.0027 | 0.7495 | 0.0478 |
| miR-8110 | -0.62985 | 0.0379 | 0.6823 | 0.0006 |

**Table S3. Selection of miRNAs from the microarray for the validation of the technique.**

| **microRNA** | **Function** | **Reference** |
| --- | --- | --- |
| **miR-29c-3p** | Regulation of insulin stimulated-glucose metabolism.  Fatty acid oxidation. | Massart J, Sjögren RJO, Lundell LS, Mudry JM, Franck N, O’Gorman DJ, Egan B, Zierath JR & Krook A (2017) Altered miR-29 Expression in Type 2 Diabetes Influences Glucose and Lipid Metabolism in Skeletal Muscle. Diabetes 66, 1807–1818.  Vienberg S, Geiger J, Madsen S & Dalgaard LT (2017) MicroRNAs in metabolism. Acta Physiol (Oxf) 219, 346–361. |
| **miR-30a-5p** | Glucose metabolism and adipogenesis. | Arias N, Aguirre L, Fernández-Quintela A, González M, Lasa A, Miranda J, Macarulla MT & Portillo MP (2016) MicroRNAs involved in the browning process of adipocytes. J Physiol Biochem 72, 509–21.  Kirby TJ, Walton RG, Finlin B, Zhu B, Unal R, Rasouli N, Peterson CA & Kern PA (2016) Integrative mRNA-microRNA analyses reveal novel interactions related to insulin sensitivity in human adipose tissue. Physiol Genomics 48, 145–53.  Koh E-H, Chernis N, Saha PK, Xiao L, Bader DA, Zhu B, Rajapakshe K, Hamilton MP, Liu X, Perera D, Chen X, York B, Trauner M, Coarfa C, Bajaj M, Moore DD, Deng T, McGuire SE & Hartig SM (2018) miR-30a Remodels Subcutaneous Adipose Tissue Inflammation to Improve Insulin Sensitivity in Obesity. Diabetes 67, 2541–2553. |
| **miR-103-3p**  **miR-107-3p** | Glucose tolerance and insulin sensitivity.  Type 2 diabetes development. | Ashoori MR, Rahmati-Yamchi M, Ostadrahimi A, Fekri Aval S & Zarghami N (2017) MicroRNAs and adipocytokines: Promising biomarkers for pharmacological targets in diabetes mellitus and its complications. Biomed Pharmacother 93, 1326–1336.  Trajkovski M, Hausser J, Soutschek J, Bhat B, Akin A, Zavolan M, Heim MH & Stoffel M (2011) MicroRNAs 103 and 107 regulate insulin sensitivity. Nature 474, 649–53.  Vienberg S, Geiger J, Madsen S & Dalgaard LT (2017) MicroRNAs in metabolism. Acta Physiol (Oxf) 219, 346–361. |
| **miR-126a-3p** | Insulin signaling pathway. | Fernandez-Twinn DS, Alfaradhi MZ, Martin-Gronert MS, Duque-Guimaraes DE, Piekarz A, Ferland-McCollough D, Bushell M & Ozanne SE (2014) Downregulation of IRS-1 in adipose tissue of offspring of obese mice is programmed cell-autonomously through post-transcriptional mechanisms. Mol Metab 3, 325–33.  Rezk NA, Sabbah NA & Saad MSS (2016) Role of MicroRNA 126 in screening, diagnosis, and prognosis of diabetic patients in Egypt. IUBMB Life 68, 452–8.  Zampetaki A, Kiechl S, Drozdov I, Willeit P, Mayr U, Prokopi M, Mayr A, Weger S, Oberhollenzer F, Bonora E, Shah A, Willeit J & Mayr M (2010) Plasma microRNA profiling reveals loss of endothelial miR-126 and other microRNAs in type 2 diabetes. Circ Res 107, 810–7. |

**Table S4. Enriched KEGG and REACTOME pathways for the genes targeted by miRNAs differentially expressed between 3m and 12m animals.**

| **Term** | **P-value** | **Adjusted P-value** | **Old P-value** | **Old Adjusted P-value** | **Z score** | **Combined Score** | **Genes** |
| --- | --- | --- | --- | --- | --- | --- | --- |
| **KEGG Pathways** |  |  |  |  |  |  |  |
| **Longevity regulating pathway - multiple species_hsa04213** | 1,52E-04 | 2,28E-02 | 3,32E-04 | 4,98E-02 | -2,02E+00 | 1,77E+01 | ADCY9; CAT; EIF4EBP2; PIK3R2 |
| Platelet activation_hsa04611 | 1,75E-03 | 9,10E-02 | 3,33E-03 | 1,64E-01 | -1,89E+00 | 1,20E+01 | ADCY9; STIM1; PIK3R2; ACTG1 |
| Chronic myeloid leukemia_hsa05220 | 3,60E-03 | 9,10E-02 | 6,16E-03 | 1,64E-01 | -1,87E+00 | 1,05E+01 | MECOM; PIK3R2; CBL |
| cGMP-PKG signaling pathway_hsa04022 | 5,39E-03 | 9,10E-02 | 9,76E-03 | 1,64E-01 | -1,81E+00 | 9,47E+00 | ADCY9; PIK3R2; SLC25A4; ADRA2A |
| Pathways in cancer _hsa05200 | 6,52E-03 | 9,10E-02 | 1,33E-02 | 1,67E-01 | -1,87E+00 | 9,41E+00 | ADCY9; RAD51; MECOM; PIK3R2; CBL; GLI3 |
| Bacterial invasion of epithelial cells_hsa05100 | 4,33E-03 | 9,10E-02 | 7,35E-03 | 1,64E-01 | -1,70E+00 | 9,24E+00 | PIK3R2; CBL; ACTG1 |
| ErbB signaling pathway_hsa04012 | 5,88E-03 | 9,10E-02 | 9,82E-03 | 1,64E-01 | -1,66E+00 | 8,54E+00 | ERBB3; PIK3R2; CBL |
| Calcium signaling pathway_hsa04020 | 7,00E-03 | 9,10E-02 | 1,25E-02 | 1,67E-01 | -1,71E+00 | 8,46E+00 | ADCY9; STIM1; ERBB3; SLC25A4 |
| Phototransduction _hsa04744 | 5,71E-03 | 9,10E-02 | 8,95E-03 | 1,64E-01 | -1,57E+00 | 8,09E+00 | GRK1; RHO |
| Longevity regulating pathway - mammal_hsa04211 | 7,28E-03 | 9,10E-02 | 1,20E-02 | 1,67E-01 | -1,60E+00 | 7,89E+00 | ADCY9; CAT; PIK3R2 |
| Proteoglycans in cancer _hsa05205 | 1,06E-02 | 1,22E-01 | 1,86E-02 | 1,99E-01 | -1,70E+00 | 7,75E+00 | ERBB3; PIK3R2; CBL; ACTG1 |
| Glycosylphosphatidylinositol(GPI)-anchor biosynthesis_ hsa00563 | 4,91E-03 | 9,10E-02 | 7,79E-03 | 1,64E-01 | -1,28E+00 | 6,78E+00 | PIGS; PIGW |
| Thyroid hormone signaling pathway_hsa04919 | 1,35E-02 | 1,26E-01 | 2,17E-02 | 2,03E-01 | -1,49E+00 | 6,40E+00 | NOTCH4; PIK3R2; ACTG1 |
| Dorso-ventral axis formation_hsa04320 | 5,71E-03 | 9,10E-02 | 8,95E-03 | 1,64E-01 | -1,20E+00 | 6,19E+00 | NOTCH4; ETS2 |
| Leukocyte transendothelial migration_hsa04670 | 1,35E-02 | 1,26E-01 | 2,17E-02 | 2,03E-01 | -1,34E+00 | 5,78E+00 | ESAM; PIK3R2; ACTG1 |
| FoxO signaling pathway_hsa04068 | 1,85E-02 | 1,54E-01 | 2,94E-02 | 2,33E-01 | -1,42E+00 | 5,66E+00 | CAT; PIK3R2; NLK |
| Tryptophan metabolism_hsa00380 | 1,23E-02 | 1,26E-01 | 1,82E-02 | 1,99E-01 | -1,26E+00 | 5,56E+00 | CAT; CYP1A2 |
| HTLV-I infection_hsa05166 | 2,34E-02 | 1,54E-01 | 3,96E-02 | 2,62E-01 | -1,42E+00 | 5,33E+00 | ADCY9; PIK3R2; SLC25A4; ETS2 |
| Endocytosis_hsa04144 | 2,37E-02 | 1,54E-01 | 4,01E-02 | 2,62E-01 | -1,42E+00 | 5,32E+00 | GRK1; ERBB3; CBL; SNX5 |
| Oxytocin signaling pathway_hsa04921 | 2,89E-02 | 1,81E-01 | 4,50E-02 | 2,70E-01 | -1,37E+00 | 4,85E+00 | ADCY9; PIK3R2; ACTG1 |
| Vibrio cholerae infection_hsa05110 | 1,95E-02 | 1,54E-01 | 2,82E-02 | 2,33E-01 | -1,20E+00 | 4,74E+00 | ADCY9; ACTG1 |
| Regulation of lipolysis in adipocytes_hsa04923 | 2,32E-02 | 1,54E-01 | 3,33E-02 | 2,38E-01 | -1,16E+00 | 4,38E+00 | ADCY9; PIK3R2 |
| MicroRNAs in cancer_hsa05206 | 3,65E-02 | 2,10E-01 | 6,05E-02 | 3,36E-01 | -1,30E+00 | 4,32E+00 | ERBB3; NOTCH4; SPRY2; PIK3R2 |
| Pathogenic Escherichia coli infection_hsa05130 | 2,24E-02 | 1,54E-01 | 3,23E-02 | 2,38E-01 | -1,07E+00 | 4,05E+00 | TUBB4A; ACTG1 |
| Pancreatic cancer_hsa05212 | 3,14E-02 | 1,89E-01 | 4,45E-02 | 2,70E-01 | -1,12E+00 | 3,89E+00 | RAD51; PIK3R2 |
| Chemokine signaling pathway_hsa04062 | 4,42E-02 | 2,37E-01 | 6,73E-02 | 3,61E-01 | -1,23E+00 | 3,83E+00 | GRK1; ADCY9; PIK3R2 |
| Adherens junction_hsa04520 | 3,87E-02 | 2,15E-01 | 5,44E-02 | 3,14E-01 | -9,97E-01 | 3,24E+00 | NLK; ACTG1 |
| Caffeine metabolism_hsa00232 | 2,08E-02 | 1,54E-01 | 2,95E-02 | 2,33E-01 | 2,63E+00 | -1,02E+01 | CYP1A2 |
|  |  |  |  |  |  |  |  |
| **REACTOME Pathways** |  |  |  |  |  |  |  |
| Spry regulation of FGF signaling_R-HSA-1295596 | 2,01E-03 | 2,19E-01 | 3,54E-03 | 3,65E-01 | -2,11E+00 | 1,31E+01 | SPRY2; CBL |
| Regulation of KIT signaling_R-HSA-1433559 | 2,01E-03 | 2,19E-01 | 3,54E-03 | 3,65E-01 | -2,09E+00 | 1,30E+01 | CBL; SH2B3 |
| Regulation of signaling by CBL_R-HSA-912631 | 2,55E-03 | 2,19E-01 | 4,37E-03 | 3,65E-01 | -2,06E+00 | 1,23E+01 | PIK3R2; CBL |
| Interleukin-7 signaling_R-HSA-1266695 | 9,35E-04 | 2,19E-01 | 1,83E-03 | 3,65E-01 | -1,70E+00 | 1,18E+01 | IL7; PIK3R2 |
| Negative regulation of FGFR3 signaling_R-HSA-5654732 | 6,57E-03 | 2,20E-01 | 1,03E-02 | 3,65E-01 | -2,24E+00 | 1,12E+01 | SPRY2; CBL |
| Post-translational modification: synthesis of GPI-anchored proteins_R-HSA-163125 | 4,91E-03 | 2,20E-01 | 7,90E-03 | 3,65E-01 | -2,06E+00 | 1,10E+01 | PIGS; PIGW |
| Negative regulation of FGFR4 signaling_R-HSA-5654733 | 7,49E-03 | 2,20E-01 | 1,17E-02 | 3,65E-01 | -2,21E+00 | 1,08E+01 | SPRY2; CBL |
| Negative regulation of FGFR1 signaling_R-HSA-5654726 | 7,97E-03 | 2,20E-01 | 1,23E-02 | 3,65E-01 | -2,24E+00 | 1,08E+01 | SPRY2; CBL |
| Signaling by FGFR4_R-HSA-5654743 | 1,30E-02 | 2,46E-01 | 2,54E-02 | 3,67E-01 | -2,48E+00 | 1,08E+01 | ADCY9; ERBB3; SPRY2; PIK3R2; CBL |
| Signaling by FGFR3_R-HSA-5654741 | 1,31E-02 | 2,46E-01 | 2,57E-02 | 3,67E-01 | -2,47E+00 | 1,07E+01 | ADCY9; ERBB3; SPRY2; PIK3R2; CBL |
| Signaling by FGFR1_R-HSA-5654736 | 1,36E-02 | 2,46E-01 | 2,66E-02 | 3,67E-01 | -2,46E+00 | 1,06E+01 | ADCY9; ERBB3; SPRY2; PIK3R2; CBL |
| Negative regulation of FGFR2 signaling_R-HSA-5654727 | 8,96E-03 | 2,20E-01 | 1,38E-02 | 3,65E-01 | -2,18E+00 | 1,03E+01 | SPRY2; CBL |
| Signaling by EGFR_R-HSA-177929 | 1,69E-02 | 2,46E-01 | 3,26E-02 | 3,67E-01 | -2,43E+00 | 9,90E+00 | ADCY9; ERBB3; SPRY2; PIK3R2; CBL |
| Signaling by FGFR2_R-HSA-5654738 | 1,80E-02 | 2,46E-01 | 3,46E-02 | 3,67E-01 | -2,46E+00 | 9,86E+00 | ADCY9; ERBB3; SPRY2; PIK3R2; CBL |
| EGFR downregulation_R-HSA-182971 | 5,71E-03 | 2,20E-01 | 9,07E-03 | 3,65E-01 | -1,90E+00 | 9,82E+00 | SPRY2; CBL |
| Inactivation, recovery and regulation of the phototransduction cascade_R-HSA-2514859 | 8,46E-03 | 2,20E-01 | 1,30E-02 | 3,65E-01 | -2,03E+00 | 9,69E+00 | GRK1; RHO |
| Signaling by FGFR_R-HSA-190236 | 1,90E-02 | 2,46E-01 | 3,64E-02 | 3,67E-01 | -2,43E+00 | 9,62E+00 | ADCY9; ERBB3; SPRY2; PIK3R2; CBL |
| The phototransduction cascade_R-HSA-2514856 | 8,96E-03 | 2,20E-01 | 1,38E-02 | 3,65E-01 | -1,99E+00 | 9,38E+00 | GRK1; RHO |
| Signaling by the B Cell Receptor (BCR)_R-HSA-983705 | 1,68E-02 | 2,46E-01 | 2,99E-02 | 3,67E-01 | -2,29E+00 | 9,37E+00 | STIM1; ERBB3; PIK3R2; CBL |
| Transport of glucose and other sugars, bile salts and organic acids, metal ions and amine compounds_R-HSA-425366 | 8,86E-03 | 2,20E-01 | 1,49E-02 | 3,65E-01 | -1,88E+00 | 8,89E+00 | SLC22A6; BSG; SLC2A6 |
| Transmembrane transport of small molecules_R-HSA-382551 | 1,24E-02 | 2,46E-01 | 2,80E-02 | 3,67E-01 | -2,01E+00 | 8,83E+00 | UNC80; SLC22A6; ADCY9; SLC20A1; ABCC5; BSG; SLC2A6 |
| Cell surface interactions at the vascular wall_R-HSA-202733 | 8,86E-03 | 2,20E-01 | 1,49E-02 | 3,65E-01 | -1,86E+00 | 8,77E+00 | BSG; ESAM; PIK3R2 |
| Integration of energy metabolism_R-HSA-163685 | 1,12E-02 | 2,46E-01 | 1,86E-02 | 3,67E-01 | -1,94E+00 | 8,71E+00 | ADCY9; SLC25A4; ADRA2A |
| G alpha (z) signalling events_R-HSA-418597 | 1,60E-02 | 2,46E-01 | 2,37E-02 | 3,67E-01 | -1,87E+00 | 7,72E+00 | ADCY9; ADRA2A |
| Disease_R-HSA-1643685 | 3,27E-02 | 2,71E-01 | 6,87E-02 | 3,67E-01 | -2,04E+00 | 6,99E+00 | ADAMTS15; MSRA; ERBB3; NOTCH4; PIK3R2; CBL; SLC25A4 |
| Antigen activates B Cell Receptor (BCR) leading to generation of second messengers_R-HSA-983695 | 1,67E-02 | 2,46E-01 | 2,47E-02 | 3,67E-01 | -1,70E+00 | 6,95E+00 | STIM1; CBL |
| Hemostasis_R-HSA-109582 | 2,86E-02 | 2,62E-01 | 5,72E-02 | 3,67E-01 | -1,87E+00 | 6,64E+00 | STIM1; BSG; ESAM; PIK3R2; SH2B3; ADRA2A |
| SLC-mediated transmembrane transport_R-HSA-425407 | 2,64E-02 | 2,62E-01 | 4,59E-02 | 3,67E-01 | -1,79E+00 | 6,51E+00 | SLC22A6; SLC20A1; BSG; SLC2A6 |
| Signaling by SCF-KIT_R-HSA-1433557 | 4,80E-02 | 2,85E-01 | 8,05E-02 | 3,68E-01 | -2,07E+00 | 6,29E+00 | ERBB3; PIK3R2; CBL; SH2B3 |
| Constitutive Signaling by Aberrant PI3K in Cancer_R-HSA-2219530 | 2,72E-02 | 2,62E-01 | 3,92E-02 | 3,67E-01 | -1,65E+00 | 5,93E+00 | ERBB3; PIK3R2 |
| Signaling by PTK6_Homo sapiens_R-HSA-8848021 | 3,23E-02 | 2,71E-01 | 4,63E-02 | 3,67E-01 | -1,67E+00 | 5,72E+00 | ERBB3; CBL |
| Diseases associated with O-glycosylation of proteins_R-HSA-3906995 | 2,80E-02 | 2,62E-01 | 4,04E-02 | 3,67E-01 | -1,57E+00 | 5,60E+00 | ADAMTS15; NOTCH4 |
| **PI5P, PP2A and IER3 Regulate PI3K/AKT Signaling_R-HSA-6811558** | 4,77E-02 | 2,85E-01 | 6,70E-02 | 3,67E-01 | -1,61E+00 | 4,91E+00 | ERBB3; PIK3R2 |
| **Regulation of insulin secretion_R-HSA-422356** | 4,46E-02 | 2,85E-01 | 6,29E-02 | 3,67E-01 | -1,51E+00 | 4,71E+00 | SLC25A4; ADRA2A |
| PTK6 Regulates RTKs and Their Effectors AKT1 and DOK1_R-HSA-8849469 | 3,72E-02 | 2,71E-01 | 4,88E-02 | 3,67E-01 | -6,18E-02 | 2,03E-01 | CBL |
| Interleukin-6 signaling_R-HSA-1059683 | 4,53E-02 | 2,85E-01 | 5,83E-02 | 3,67E-01 | 1,30E-01 | -4,01E-01 | CBL |
| Signaling by NOTCH4_R-HSA-1980150 | 4,53E-02 | 2,85E-01 | 5,83E-02 | 3,67E-01 | 1,94E-01 | -6,02E-01 | NOTCH4 |
| A third proteolytic cleavage releases NICD_R-HSA-157212 | 3,72E-02 | 2,71E-01 | 4,88E-02 | 3,67E-01 | 2,26E-01 | -7,43E-01 | NOTCH4 |
| GRB7 events in ERBB2 signaling_R-HSA-1306955 | 2,08E-02 | 2,46E-01 | 2,96E-02 | 3,67E-01 | 2,17E-01 | -8,41E-01 | ERBB3 |
| Synthesis of (16-20)-hydroxyeicosatetraenoic acids (HETE)_R-HSA-2142816 | 3,72E-02 | 2,71E-01 | 4,88E-02 | 3,67E-01 | 3,70E-01 | -1,22E+00 | CYP1A2 |
| Organic anion transport_R-HSA-561048 | 2,08E-02 | 2,46E-01 | 2,96E-02 | 3,67E-01 | 3,26E-01 | -1,26E+00 | SLC22A6 |
| Opsins_R-HSA-419771 | 3,72E-02 | 2,71E-01 | 4,88E-02 | 3,67E-01 | 4,01E-01 | -1,32E+00 | RHO |
| Adenylate cyclase activating pathway_R-HSA-170660 | 4,12E-02 | 2,85E-01 | 5,36E-02 | 3,67E-01 | 4,14E-01 | -1,32E+00 | ADCY9 |
| Activation of the phototransduction cascade_R-HSA-2485179 | 4,53E-02 | 2,85E-01 | 5,83E-02 | 3,67E-01 | 4,50E-01 | -1,39E+00 | RHO |
| Purine catabolism_R-HSA-74259 | 4,12E-02 | 2,85E-01 | 5,36E-02 | 3,67E-01 | 4,50E-01 | -1,44E+00 | CAT |
| GLI proteins bind promoters of Hh responsive genes to promote transcription_R-HSA-5635851 | 2,90E-02 | 2,62E-01 | 3,92E-02 | 3,67E-01 | 4,12E-01 | -1,46E+00 | GLI3 |
| Attachment of GPI anchor to uPAR_R-HSA-162791 | 2,90E-02 | 2,62E-01 | 3,92E-02 | 3,67E-01 | 4,31E-01 | -1,52E+00 | PIGS |
| Protein repair_R-HSA-5676934 | 2,49E-02 | 2,62E-01 | 3,44E-02 | 3,67E-01 | 4,43E-01 | -1,63E+00 | MSRA |
| Adrenoceptors_R-HSA-390696 | 3,72E-02 | 2,71E-01 | 4,88E-02 | 3,67E-01 | 5,15E-01 | -1,69E+00 | ADRA2A |
| Pre-NOTCH Processing in the Endoplasmic Reticulum_R-HSA-1912399 | 2,49E-02 | 2,62E-01 | 3,44E-02 | 3,67E-01 | 5,85E-01 | -2,16E+00 | NOTCH4 |
| Pentose phosphate pathway (hexose monophosphate shunt)_R-HSA-71336 | 3,72E-02 | 2,71E-01 | 4,88E-02 | 3,67E-01 | 6,64E-01 | -2,18E+00 | RPIA |
| Synthesis of epoxy (EET) and dihydroxyeicosatrienoic acids (DHET)_R-HSA-2142670 | 3,31E-02 | 2,71E-01 | 4,40E-02 | 3,67E-01 | 6,48E-01 | -2,21E+00 | CYP1A2 |
| Proton-coupled monocarboxylate transport_R-HSA-433692 | 2,49E-02 | 2,62E-01 | 3,44E-02 | 3,67E-01 | 6,24E-01 | -2,30E+00 | BSG |
| Sodium-coupled phosphate cotransporters_R-HSA-427652 | 2,08E-02 | 2,46E-01 | 2,96E-02 | 3,67E-01 | 7,42E-01 | -2,87E+00 | SLC20A1 |
| Hyaluronan biosynthesis and export_R-HSA-2142850 | 2,08E-02 | 2,46E-01 | 2,96E-02 | 3,67E-01 | 1,58E+00 | -6,10E+00 | ABCC5 |

**Table S5. Enriched gene ontology (GO) terms for molecular function and biological process for the genes targeted by miRNAs differentially expressed between 3m vs 12m animals.**

| **Term** | **P-value** | **Adjusted P-value** | **Old P-value** | **Old Adjusted P-value** | **Z score** | **Combined Score** | **Genes** |
| --- | --- | --- | --- | --- | --- | --- | --- |
| **GO Terms Molecular function** |  |  |  |  |  |  |  |
| Lys48-specific deubiquitinase activity (GO:1990380) | 4,80E-04 | 3,26E-02 | 9,87E-04 | 6,37E-02 | -3,94E+00 | 3,01E+01 | OTUD5; YOD1 |
| protein heterodimerization activity (GO:0046982) | 3,47E-03 | 9,21E-02 | 6,24E-03 | 1,41E-01 | -5,04E+00 | 2,86E+01 | ERBB3; NOTCH4; PIK3R2; SNX5; ADRA2A |
| ubiquitin protein ligase binding (GO:0031625) | 5,27E-03 | 1,12E-01 | 9,29E-03 | 1,41E-01 | -5,16E+00 | 2,70E+01 | ERBB3; YOD1; RFFL; NLK; ACTG1 |
| protein kinase binding (GO:0019901) | 7,75E-03 | 1,14E-01 | 1,34E-02 | 1,41E-01 | -5,28E+00 | 2,56E+01 | TRIM6; SPRY2; SMCR8; RFFL; ADRA2A |
| Lys63-specific deubiquitinase activity (GO:0061578) | 6,16E-04 | 3,26E-02 | 1,20E-03 | 6,37E-02 | -3,02E+00 | 2,23E+01 | OTUD5; YOD1 |
| organic anion transmembrane transporter activity (GO:0008514) | 3,48E-03 | 9,21E-02 | 5,33E-03 | 1,41E-01 | -2,86E+00 | 1,62E+01 | SLC22A6; ABCC5 |
| cadherin binding (GO:0045296) | 2,77E-02 | 1,14E-01 | 4,26E-02 | 1,41E-01 | -4,15E+00 | 1,49E+01 | PPME1; BSG; CBL; SNX5 |
| ubiquitin protein ligase activity (GO:0061630) | 2,24E-02 | 1,14E-01 | 3,26E-02 | 1,41E-01 | -3,83E+00 | 1,45E+01 | TRIM6; RFFL; CBL |
| protein phosphatase binding (GO:0019903) | 1,87E-02 | 1,14E-01 | 2,56E-02 | 1,41E-01 | -3,08E+00 | 1,22E+01 | PPME1; PIK3R2 |
| receptor tyrosine kinase binding (GO:0030971) | 9,48E-03 | 1,14E-01 | 1,35E-02 | 1,41E-01 | -2,59E+00 | 1,20E+01 | PIK3R2; CBL |
| heme binding (GO:0020037) | 2,40E-02 | 1,14E-01 | 3,23E-02 | 1,41E-01 | -2,96E+00 | 1,10E+01 | CYP1A2; CAT |
| thiol-dependent ubiquitin-specific protease activity (GO:0004843) | 3,41E-02 | 1,14E-01 | 4,53E-02 | 1,41E-01 | -2,55E+00 | 8,61E+00 | OTUD5; YOD1 |
| phosphatidylinositol-4,5-bisphosphate 3-kinase activity (GO:0046934) | 3,06E-02 | 1,14E-01 | 4,08E-02 | 1,41E-01 | -2,43E+00 | 8,47E+00 | ERBB3; PIK3R2 |
| structural constituent of cytoskeleton (GO:0005200) | 4,77E-02 | 1,16E-01 | 6,24E-02 | 1,47E-01 | -1,92E+00 | 5,84E+00 | SORBS2; ACTG1 |
| protease binding (GO:0002020) | 4,26E-02 | 1,14E-01 | 5,61E-02 | 1,41E-01 | -1,83E+00 | 5,79E+00 | STIM1; RFFL |
| epinephrine binding (GO:0051379) | 2,49E-02 | 1,14E-01 | 3,30E-02 | 1,41E-01 | 2,03E-01 | -7,50E-01 | ADRA2A |
| protein tyrosine kinase binding (GO:1990782) | 2,08E-02 | 1,14E-01 | 2,84E-02 | 1,41E-01 | 3,88E-01 | -1,50E+00 | TRIM6 |
| translation repressor activity (GO:0030371) | 4,12E-02 | 1,14E-01 | 5,14E-02 | 1,41E-01 | 5,01E-01 | -1,60E+00 | EIF4EBP2 |
| single-stranded DNA-dependent ATPase activity (GO:0043142) | 4,12E-02 | 1,14E-01 | 5,14E-02 | 1,41E-01 | 5,94E-01 | -1,89E+00 | RAD51 |
| protein tyrosine kinase activator activity (GO:0030296) | 2,90E-02 | 1,14E-01 | 3,77E-02 | 1,41E-01 | 6,21E-01 | -2,20E+00 | ERBB3 |
| 1-phosphatidylinositol-3-kinase regulator activity (GO:0046935) | 2,49E-02 | 1,14E-01 | 3,30E-02 | 1,41E-01 | 6,73E-01 | -2,48E+00 | PIK3R2 |
| urate transmembrane transporter activity (GO:0015143) | 4,12E-02 | 1,14E-01 | 5,14E-02 | 1,41E-01 | 8,30E-01 | -2,65E+00 | SLC22A6 |
| D-glucose transmembrane transporter activity (GO:0055056) | 3,72E-02 | 1,14E-01 | 4,69E-02 | 1,41E-01 | 8,49E-01 | -2,80E+00 | SLC2A6 |
| recombinase activity (GO:0000150) | 3,31E-02 | 1,14E-01 | 4,23E-02 | 1,41E-01 | 8,63E-01 | -2,94E+00 | RAD51 |
| eukaryotic initiation factor 4E binding (GO:0008190) | 4,12E-02 | 1,14E-01 | 5,14E-02 | 1,41E-01 | 9,39E-01 | -2,99E+00 | EIF4EBP2 |
| ADP transmembrane transporter activity (GO:0015217) | 2,49E-02 | 1,14E-01 | 3,30E-02 | 1,41E-01 | 8,49E-01 | -3,13E+00 | SLC25A23 |
| basic amino acid transmembrane transporter activity (GO:0015174) | 2,90E-02 | 1,14E-01 | 3,77E-02 | 1,41E-01 | 9,15E-01 | -3,24E+00 | PQLC2 |
| cytoskeletal adaptor activity (GO:0008093) | 3,72E-02 | 1,14E-01 | 4,69E-02 | 1,41E-01 | 1,02E+00 | -3,35E+00 | SORBS2 |
| oxidoreductase activity, acting on paired donors, with incorporation or reduction of molecular oxygen, reduced flavin or flavoprotein as one donor, and incorporation of one atom of oxygen (GO:0016712) | 2,08E-02 | 1,14E-01 | 2,84E-02 | 1,41E-01 | 8,73E-01 | -3,38E+00 | CYP1A2 |
| MAP kinase activity (GO:0004707) | 4,53E-02 | 1,14E-01 | 5,60E-02 | 1,41E-01 | 1,12E+00 | -3,48E+00 | NLK |
| sodium:phosphate symporter activity (GO:0005436) | 3,72E-02 | 1,14E-01 | 4,69E-02 | 1,41E-01 | 1,15E+00 | -3,77E+00 | SLC20A1 |
| monocarboxylic acid transmembrane transporter activity (GO:0008028) | 4,12E-02 | 1,14E-01 | 5,14E-02 | 1,41E-01 | 1,18E+00 | -3,77E+00 | BSG |
| thioesterase binding (GO:0031996) | 4,53E-02 | 1,14E-01 | 5,60E-02 | 1,41E-01 | 1,32E+00 | -4,10E+00 | ADRA2A |
| extracellular matrix constituent conferring elasticity (GO:0030023) | 2,08E-02 | 1,14E-01 | 2,84E-02 | 1,41E-01 | 1,10E+00 | -4,24E+00 | EMILIN2 |
| Rac guanyl-nucleotide exchange factor activity (GO:0030676) | 4,93E-02 | 1,16E-01 | 6,05E-02 | 1,46E-01 | 1,42E+00 | -4,28E+00 | FARP1 |
| ATPase-coupled anion transmembrane transporter activity (GO:0043225) | 4,53E-02 | 1,14E-01 | 5,60E-02 | 1,41E-01 | 1,39E+00 | -4,30E+00 | ABCC5 |
| ATP transmembrane transporter activity (GO:0005347) | 2,49E-02 | 1,14E-01 | 3,30E-02 | 1,41E-01 | 1,22E+00 | -4,52E+00 | SLC25A23 |
| RNA helicase activity (GO:0003724) | 4,12E-02 | 1,14E-01 | 5,14E-02 | 1,41E-01 | 1,43E+00 | -4,57E+00 | DDX10 |
| microtubule plus-end binding (GO:0051010) | 4,53E-02 | 1,14E-01 | 5,60E-02 | 1,41E-01 | 1,48E+00 | -4,58E+00 | STIM1 |
| sodium-dependent phosphate transmembrane transporter activity (GO:0015321) | 2,90E-02 | 1,14E-01 | 3,77E-02 | 1,41E-01 | 1,34E+00 | -4,73E+00 | SLC20A1 |
| protein serine/threonine kinase inhibitor activity (GO:0030291) | 3,31E-02 | 1,14E-01 | 4,23E-02 | 1,41E-01 | 1,40E+00 | -4,77E+00 | SPRY2 |
| sugar:proton symporter activity (GO:0005351) | 2,90E-02 | 1,14E-01 | 3,77E-02 | 1,41E-01 | 1,38E+00 | -4,88E+00 | SLC2A6 |
| scavenger receptor activity (GO:0005044) | 4,93E-02 | 1,16E-01 | 6,05E-02 | 1,46E-01 | 1,80E+00 | -5,41E+00 | SCARA3 |
| dynactin binding (GO:0034452) | 4,12E-02 | 1,14E-01 | 5,14E-02 | 1,41E-01 | 1,73E+00 | -5,53E+00 | SNX5 |
| O-acyltransferase activity (GO:0008374) | 2,49E-02 | 1,14E-01 | 3,30E-02 | 1,41E-01 | 1,58E+00 | -5,84E+00 | PIGW |
| **GO Terms Biological Process** |  |  |  |  |  |  |  |
| UV protection (GO:0009650) | 7,67E-04 | 1,37E-01 | 1,18E-03 | 1,54E-01 | -3,96E+00 | 2,84E+01 | SCARA3; CAT |
| rhodopsin mediated signaling pathway (GO:0016056) | 9,35E-04 | 1,37E-01 | 1,39E-03 | 1,54E-01 | -2,97E+00 | 2,07E+01 | GRK1; RHO |
| adenylate cyclase-activating adrenergic receptor signaling pathway (GO:0071880) | 2,55E-03 | 1,39E-01 | 3,32E-03 | 1,54E-01 | -2,81E+00 | 1,68E+01 | ADCY9; ADRA2A |
| protein K48-linked deubiquitination (GO:0071108) | 2,85E-03 | 1,39E-01 | 3,66E-03 | 1,54E-01 | -2,59E+00 | 1,52E+01 | OTUD5; YOD1 |
| regulation of rhodopsin mediated signaling pathway (GO:0022400) | 4,91E-03 | 1,39E-01 | 6,02E-03 | 1,54E-01 | -2,65E+00 | 1,41E+01 | GRK1; RHO |
| protein K63-linked deubiquitination (GO:0070536) | 3,81E-03 | 1,39E-01 | 4,77E-03 | 1,54E-01 | -2,39E+00 | 1,33E+01 | OTUD5; YOD1 |
| phosphatidylinositol 3-kinase signaling (GO:0014065) | 5,31E-03 | 1,39E-01 | 6,46E-03 | 1,54E-01 | -2,41E+00 | 1,26E+01 | ERBB3; PIK3R2 |
| negative regulation of epidermal growth factor receptor signaling pathway (GO:0042059) | 6,57E-03 | 1,39E-01 | 7,88E-03 | 1,54E-01 | -2,07E+00 | 1,04E+01 | SPRY2; CBL |
| regulation of autophagy (GO:0010506) | 1,41E-02 | 1,39E-01 | 1,61E-02 | 1,54E-01 | -2,42E+00 | 1,03E+01 | SMCR8; PIK3R2 |
| positive regulation of phosphatidylinositol 3-kinase signaling (GO:0014068) | 1,95E-02 | 1,39E-01 | 2,20E-02 | 1,54E-01 | -2,39E+00 | 9,41E+00 | ERBB3; CBL |
| regulation of insulin secretion (GO:0050796) | 2,40E-02 | 1,39E-01 | 2,68E-02 | 1,54E-01 | -2,22E+00 | 8,29E+00 | SLC25A4; ADRA2A |
| positive regulation of peptidyl-serine phosphorylation (GO:0033138) | 2,40E-02 | 1,39E-01 | 2,68E-02 | 1,54E-01 | -2,08E+00 | 7,77E+00 | TRIM6; SPRY2 |
| negative regulation of apoptotic process (GO:0043066) | 4,71E-02 | 1,39E-01 | 5,27E-02 | 1,54E-01 | -2,37E+00 | 7,24E+00 | IL7; CAT; SPRY2; CBL |
| vascular endothelial growth factor receptor signaling pathway (GO:0048010) | 3,32E-02 | 1,39E-01 | 3,68E-02 | 1,54E-01 | -1,38E+00 | 4,70E+00 | PIK3R2; ACTG1 |
| transforming growth factor beta receptor signaling pathway (GO:0007179) | 3,97E-02 | 1,39E-01 | 4,37E-02 | 1,54E-01 | -1,16E+00 | 3,73E+00 | CBL; NLK |
| regulation of gene expression (GO:0010468) | 3,78E-02 | 1,39E-01 | 4,17E-02 | 1,54E-01 | -1,05E+00 | 3,43E+00 | TOB2; NLK |
| response to lipopolysaccharide (GO:0032496) | 4,26E-02 | 1,39E-01 | 4,68E-02 | 1,54E-01 | -1,06E+00 | 3,35E+00 | OTUD5; TRIM6 |
| regulation of phosphatidylinositol 3-kinase signaling (GO:0014066) | 4,36E-02 | 1,39E-01 | 4,79E-02 | 1,54E-01 | -1,04E+00 | 3,24E+00 | ERBB3; PIK3R2 |
| movement of cell or subcellular component (GO:0006928) | 4,46E-02 | 1,39E-01 | 4,89E-02 | 1,54E-01 | -9,97E-01 | 3,10E+00 | ADRA2A; ACTG1 |
| methionine metabolic process (GO:0006555) | 2,08E-02 | 1,39E-01 | 2,57E-02 | 1,54E-01 | 2,51E-01 | -9,70E-01 | MSRA |
| mRNA 3'-splice site recognition (GO:0000389) | 2,49E-02 | 1,39E-01 | 2,99E-02 | 1,54E-01 | 8,56E-01 | -3,16E+00 | SF1 |
| monoterpenoid metabolic process (GO:0016098) | 2,49E-02 | 1,39E-01 | 2,99E-02 | 1,54E-01 | 8,63E-01 | -3,19E+00 | CYP1A2 |
| protein repair (GO:0030091) | 2,49E-02 | 1,39E-01 | 2,99E-02 | 1,54E-01 | 9,80E-01 | -3,62E+00 | MSRA |
| hematopoietic stem cell differentiation (GO:0060218) | 2,49E-02 | 1,39E-01 | 2,99E-02 | 1,54E-01 | 1,15E+00 | -4,25E+00 | HOXB4 |
| positive regulation of potassium ion transport (GO:0043268) | 2,90E-02 | 1,39E-01 | 3,41E-02 | 1,54E-01 | 1,24E+00 | -4,38E+00 | ADRA2A |
| regulation of NIK/NF-kappaB signaling (GO:1901222) | 2,49E-02 | 1,39E-01 | 2,99E-02 | 1,54E-01 | 1,22E+00 | -4,51E+00 | TRIM6 |
| drug catabolic process (GO:0042737) | 2,08E-02 | 1,39E-01 | 2,57E-02 | 1,54E-01 | 1,23E+00 | -4,77E+00 | CYP1A2 |
| morphogenesis of a branching structure (GO:0001763) | 2,08E-02 | 1,39E-01 | 2,57E-02 | 1,54E-01 | 1,24E+00 | -4,80E+00 | NOTCH4 |
| oxidative demethylation (GO:0070989) | 3,72E-02 | 1,39E-01 | 4,24E-02 | 1,54E-01 | 1,48E+00 | -4,88E+00 | CYP1A2 |
| intestinal absorption (GO:0050892) | 2,90E-02 | 1,39E-01 | 3,41E-02 | 1,54E-01 | 1,40E+00 | -4,96E+00 | ADRA2A |
| regulation of cellular protein metabolic process (GO:0032268) | 2,08E-02 | 1,39E-01 | 2,57E-02 | 1,54E-01 | 1,38E+00 | -5,34E+00 | SSH1 |
| omega-hydroxylase P450 pathway (GO:0097267) | 3,72E-02 | 1,39E-01 | 4,24E-02 | 1,54E-01 | 1,87E+00 | -6,14E+00 | CYP1A2 |
| modulation of synaptic transmission (GO:0050804) | 4,12E-02 | 1,39E-01 | 4,65E-02 | 1,54E-01 | 1,95E+00 | -6,23E+00 | EIF4EBP2 |
| negative regulation of T cell activation (GO:0050868) | 3,31E-02 | 1,39E-01 | 3,82E-02 | 1,54E-01 | 1,88E+00 | -6,40E+00 | TNFAIP8L2 |
| regulation of mitochondrial membrane permeability (GO:0046902) | 4,12E-02 | 1,39E-01 | 4,65E-02 | 1,54E-01 | 2,02E+00 | -6,44E+00 | SLC25A4 |
| response to metal ion (GO:0010038) | 4,12E-02 | 1,39E-01 | 4,65E-02 | 1,54E-01 | 2,09E+00 | -6,68E+00 | TESMIN |
| T cell differentiation in thymus (GO:0033077) | 4,12E-02 | 1,39E-01 | 4,65E-02 | 1,54E-01 | 2,16E+00 | -6,89E+00 | GLI3 |
| cellular response to ATP (GO:0071318) | 3,72E-02 | 1,39E-01 | 4,24E-02 | 1,54E-01 | 2,12E+00 | -6,96E+00 | SSH1 |
| 5S class rRNA transcription from RNA polymerase III type 1 promoter (GO:0042791) | 2,49E-02 | 1,39E-01 | 2,99E-02 | 1,54E-01 | 2,02E+00 | -7,44E+00 | GTF3C2 |
| negative regulation of epidermal growth factor-activated receptor activity (GO:0007175) | 3,72E-02 | 1,39E-01 | 4,24E-02 | 1,54E-01 | 2,29E+00 | -7,54E+00 | CBL |
| positive regulation of T cell differentiation (GO:0045582) | 2,90E-02 | 1,39E-01 | 3,41E-02 | 1,54E-01 | 2,14E+00 | -7,57E+00 | IL7 |
| glucose import (GO:0046323) | 3,72E-02 | 1,39E-01 | 4,24E-02 | 1,54E-01 | 2,30E+00 | -7,58E+00 | SLC2A6 |
| extrinsic apoptotic signaling pathway in absence of ligand (GO:0097192) | 4,12E-02 | 1,39E-01 | 4,65E-02 | 1,54E-01 | 2,38E+00 | -7,60E+00 | ERBB3 |
| positive regulation of type I interferon-mediated signaling pathway (GO:0060340) | 2,49E-02 | 1,39E-01 | 2,99E-02 | 1,54E-01 | 2,07E+00 | -7,65E+00 | TRIM6 |
| protein K11-linked deubiquitination (GO:0035871) | 4,12E-02 | 1,39E-01 | 4,65E-02 | 1,54E-01 | 2,41E+00 | -7,69E+00 | YOD1 |
| protein trimerization (GO:0070206) | 2,90E-02 | 1,39E-01 | 3,41E-02 | 1,54E-01 | 2,24E+00 | -7,91E+00 | TRIM6 |
| tRNA transcription from RNA polymerase III promoter (GO:0042797) | 2,49E-02 | 1,39E-01 | 2,99E-02 | 1,54E-01 | 2,14E+00 | -7,91E+00 | GTF3C2 |
| negative regulation of calcium ion transport (GO:0051926) | 3,31E-02 | 1,39E-01 | 3,82E-02 | 1,54E-01 | 2,33E+00 | -7,93E+00 | ADRA2A |
| heterocycle metabolic process (GO:0046483) | 2,49E-02 | 1,39E-01 | 2,99E-02 | 1,54E-01 | 2,15E+00 | -7,94E+00 | CYP1A2 |
| response to reactive oxygen species (GO:0000302) | 3,72E-02 | 1,39E-01 | 4,24E-02 | 1,54E-01 | 2,43E+00 | -7,99E+00 | CAT |
| embryonic digestive tract development (GO:0048566) | 4,53E-02 | 1,39E-01 | 5,07E-02 | 1,54E-01 | 2,66E+00 | -8,23E+00 | GLI3 |
| negative regulation of microtubule polymerization (GO:0031115) | 3,31E-02 | 1,39E-01 | 3,82E-02 | 1,54E-01 | 2,44E+00 | -8,32E+00 | TUBB4A |
| mitochondrial calcium uptake (GO:0036444) | 3,72E-02 | 1,39E-01 | 4,24E-02 | 1,54E-01 | 2,55E+00 | -8,40E+00 | SLC25A23 |
| cellular metal ion homeostasis (GO:0006875) | 2,49E-02 | 1,39E-01 | 2,99E-02 | 1,54E-01 | 2,30E+00 | -8,49E+00 | TESMIN |
| positive regulation of alpha-beta T cell differentiation (GO:0046638) | 2,08E-02 | 1,39E-01 | 2,57E-02 | 1,54E-01 | 2,24E+00 | -8,66E+00 | GLI3 |
| cell junction assembly (GO:0034329) | 4,53E-02 | 1,39E-01 | 5,07E-02 | 1,54E-01 | 2,86E+00 | -8,86E+00 | ACTG1 |
| phototransduction, visible light (GO:0007603) | 2,90E-02 | 1,39E-01 | 3,41E-02 | 1,54E-01 | 2,55E+00 | -9,02E+00 | RHO |
| pentose-phosphate shunt (GO:0006098) | 4,93E-02 | 1,39E-01 | 5,48E-02 | 1,54E-01 | 3,12E+00 | -9,41E+00 | RPIA |
| telomere maintenance via telomere lengthening (GO:0010833) | 2,90E-02 | 1,39E-01 | 3,41E-02 | 1,54E-01 | 2,70E+00 | -9,55E+00 | RAD51 |
| regulation of lamellipodium assembly (GO:0010591) | 4,12E-02 | 1,39E-01 | 4,65E-02 | 1,54E-01 | 3,08E+00 | -9,82E+00 | SSH1 |
| negative regulation of autophagosome assembly (GO:1902902) | 4,12E-02 | 1,39E-01 | 4,65E-02 | 1,54E-01 | 3,11E+00 | -9,91E+00 | SMCR8 |
| negative regulation of smoothened signaling pathway (GO:0045879) | 4,53E-02 | 1,39E-01 | 5,07E-02 | 1,54E-01 | 3,44E+00 | -1,07E+01 | GLI3 |
| negative regulation of calcium ion-dependent exocytosis (GO:0045955) | 2,08E-02 | 1,39E-01 | 2,57E-02 | 1,54E-01 | 2,79E+00 | -1,08E+01 | ADRA2A |
| pentose-phosphate shunt, non-oxidative branch (GO:0009052) | 2,08E-02 | 1,39E-01 | 2,57E-02 | 1,54E-01 | 2,80E+00 | -1,08E+01 | RPIA |
| free ubiquitin chain polymerization (GO:0010994) | 2,08E-02 | 1,39E-01 | 2,57E-02 | 1,54E-01 | 3,16E+00 | -1,23E+01 | TRIM6 |
| store-operated calcium entry (GO:0002115) | 2,90E-02 | 1,39E-01 | 3,41E-02 | 1,54E-01 | 3,85E+00 | -1,36E+01 | STIM1 |
| positive regulation of transcription of Notch receptor target (GO:0007221) | 2,49E-02 | 1,39E-01 | 2,99E-02 | 1,54E-01 | 3,79E+00 | -1,40E+01 | NOTCH4 |
| detection of calcium ion (GO:0005513) | 4,12E-02 | 1,39E-01 | 4,65E-02 | 1,54E-01 | 5,10E+00 | -1,63E+01 | STIM1 |
| positive regulation of autophagosome maturation (GO:1901098) | 2,49E-02 | 1,39E-01 | 2,99E-02 | 1,54E-01 | 4,61E+00 | -1,70E+01 | SMCR8 |
| regulation of TORC1 signaling (GO:1903432) | 2,08E-02 | 1,39E-01 | 2,57E-02 | 1,54E-01 | 4,40E+00 | -1,70E+01 | SMCR8 |
| bone resorption (GO:0045453) | 3,31E-02 | 1,39E-01 | 3,82E-02 | 1,54E-01 | 5,11E+00 | -1,74E+01 | IL7 |
| negative regulation of peptidyl-threonine phosphorylation (GO:0010801) | 4,93E-02 | 1,39E-01 | 5,48E-02 | 1,54E-01 | 5,93E+00 | -1,79E+01 | SPRY2 |
| organic anion transport (GO:0015711) | 3,31E-02 | 1,39E-01 | 3,82E-02 | 1,54E-01 | 5,28E+00 | -1,80E+01 | SLC22A6 |
| negative regulation of cAMP biosynthetic process (GO:0030818) | 3,72E-02 | 1,39E-01 | 4,24E-02 | 1,54E-01 | 5,64E+00 | -1,86E+01 | ADRA2A |
| positive regulation of epidermal growth factor-activated receptor activity (GO:0045741) | 3,31E-02 | 1,39E-01 | 3,82E-02 | 1,54E-01 | 5,45E+00 | -1,86E+01 | ADRA2A |
| toll-like receptor 3 signaling pathway (GO:0034138) | 2,49E-02 | 1,39E-01 | 2,99E-02 | 1,54E-01 | 5,10E+00 | -1,88E+01 | SCARA3 |
| negative regulation of lipid catabolic process (GO:0050995) | 4,93E-02 | 1,39E-01 | 5,48E-02 | 1,54E-01 | 6,30E+00 | -1,90E+01 | ADRA2A |
| mitochondrial genome maintenance (GO:0000002) | 4,93E-02 | 1,39E-01 | 5,48E-02 | 1,54E-01 | 6,32E+00 | -1,90E+01 | SLC25A4 |
| hyaluronan biosynthetic process (GO:0030213) | 2,90E-02 | 1,39E-01 | 3,41E-02 | 1,54E-01 | 5,44E+00 | -1,93E+01 | ABCC5 |
| strand invasion (GO:0042148) | 2,90E-02 | 1,39E-01 | 3,41E-02 | 1,54E-01 | 5,55E+00 | -1,96E+01 | RAD51 |
| entry of bacterium into host cell (GO:0035635) | 4,53E-02 | 1,39E-01 | 5,07E-02 | 1,54E-01 | 6,56E+00 | -2,03E+01 | CBL |
| attachment of GPI anchor to protein (GO:0016255) | 2,90E-02 | 1,39E-01 | 3,41E-02 | 1,54E-01 | 5,77E+00 | -2,04E+01 | PIGS |
| mammary gland development (GO:0030879) | 3,72E-02 | 1,39E-01 | 4,24E-02 | 1,54E-01 | 6,26E+00 | -2,06E+01 | NOTCH4 |
| cell fate determination (GO:0001709) | 3,72E-02 | 1,39E-01 | 4,24E-02 | 1,54E-01 | 6,35E+00 | -2,09E+01 | NOTCH4 |
| protein exit from endoplasmic reticulum (GO:0032527) | 2,49E-02 | 1,39E-01 | 2,99E-02 | 1,54E-01 | 5,76E+00 | -2,12E+01 | TMED9 |
| regulation of actin polymerization or depolymerization (GO:0008064) | 2,90E-02 | 1,39E-01 | 3,41E-02 | 1,54E-01 | 6,01E+00 | -2,13E+01 | SSH1 |
| regulation of axonogenesis (GO:0050770) | 4,53E-02 | 1,39E-01 | 5,07E-02 | 1,54E-01 | 6,87E+00 | -2,13E+01 | SSH1 |
| mitotic recombination (GO:0006312) | 4,93E-02 | 1,39E-01 | 5,48E-02 | 1,54E-01 | 7,07E+00 | -2,13E+01 | RAD51 |
| hydrogen peroxide catabolic process (GO:0042744) | 4,93E-02 | 1,39E-01 | 5,48E-02 | 1,54E-01 | 7,12E+00 | -2,14E+01 | CAT |
| exogenous drug catabolic process (GO:0042738) | 4,53E-02 | 1,39E-01 | 5,07E-02 | 1,54E-01 | 6,99E+00 | -2,16E+01 | CYP1A2 |
| cellular response to interferon-beta (GO:0035458) | 4,53E-02 | 1,39E-01 | 5,07E-02 | 1,54E-01 | 7,00E+00 | -2,17E+01 | TRIM6 |
| negative regulation of translational initiation (GO:0045947) | 4,93E-02 | 1,39E-01 | 5,48E-02 | 1,54E-01 | 7,22E+00 | -2,17E+01 | EIF4EBP2 |
| positive regulation of glucose import in response to insulin stimulus (GO:2001275) | 3,31E-02 | 1,39E-01 | 3,82E-02 | 1,54E-01 | 6,45E+00 | -2,20E+01 | PIK3R2 |
| positive regulation of chondrocyte differentiation (GO:0032332) | 4,53E-02 | 1,39E-01 | 5,07E-02 | 1,54E-01 | 7,35E+00 | -2,27E+01 | SOX5 |
| urate transport (GO:0015747) | 4,12E-02 | 1,39E-01 | 4,65E-02 | 1,54E-01 | 7,19E+00 | -2,29E+01 | SLC22A6 |
| early endosome to Golgi transport (GO:0034498) | 4,93E-02 | 1,39E-01 | 5,48E-02 | 1,54E-01 | 7,79E+00 | -2,34E+01 | TMED9 |
| positive regulation of cartilage development (GO:0061036) | 4,53E-02 | 1,39E-01 | 5,07E-02 | 1,54E-01 | 7,80E+00 | -2,42E+01 | SOX5 |
| negative regulation of macroautophagy (GO:0016242) | 3,72E-02 | 1,39E-01 | 4,24E-02 | 1,54E-01 | 7,36E+00 | -2,42E+01 | SMCR8 |
| limb morphogenesis (GO:0035108) | 2,90E-02 | 1,39E-01 | 3,41E-02 | 1,54E-01 | 7,45E+00 | -2,64E+01 | GLI3 |
| negative regulation of necroptotic process (GO:0060546) | 2,49E-02 | 1,39E-01 | 2,99E-02 | 1,54E-01 | 7,26E+00 | -2,68E+01 | SLC25A4 |

**Table S6. Enriched TRANSFAC and JASPAR terms for the transcription factor genes targeted by miRNAs differentially
expressed between 3m vs 12m animals.**

| **Term** | **P-value** | **Adjusted P-value** | **Old P-value** | **Old Adjusted P-value** | **Z score** | **Combined Score** | **Genes** |
| --- | --- | --- | --- | --- | --- | --- | --- |
| GABPA (human) | 1,11E-03 | 2,69E-01 | 3,20E-04 | 2,58E-02 | -2,03E+00 | 1,38E+01 | PIK3R2; TNFAIP8L2; SLC25A23; TOB2 |
| TP53 (mouse) | 2,30E-03 | 2,79E-01 | 7,47E-05 | 1,19E-02 | -1,73E+00 | 1,05E+01 | DDX10; CBL; STIM1; BSG; GPD1; CYP1A2; IGDCC3; SPRY3; EIF4EBP2; RFFL; SNX5; SERF2; SF1 |
| NR5A2 (mouse) | 1,37E-02 | 8,44E-01 | 5,00E-04 | 3,01E-02 | -1,67E+00 | 7,16E+00 | FARP1; SLC20A1; AI464131; NOTCH4; CBL; RPIA; TMEM214; PPME1; CAT; ESAM; ZFP740; SH2B3; SNX5; SF1 |
| ZNF281 (human) | 2,24E-02 | 8,44E-01 | 2,32E-03 | 5,72E-02 | -1,79E+00 | 6,80E+00 | UNC80; TRIM6; HSPB6; BSG; HOXB4; ZKSCAN2; SH2B3; ACTG1; SSH1 |
| ZBTB7A (human) | 2,83E-02 | 8,44E-01 | 2,11E-03 | 5,72E-02 | -1,66E+00 | 5,93E+00 | SCARA3; ADCY9; RAD51; STIM1; NDST3; ZKSCAN2; ESAM; NR2E1; NLK; ZSWIM5; SNX5 |
| E2F1 (human) | 2,12E-02 | 8,44E-01 | 9,83E-05 | 1,19E-02 | -1,53E+00 | 5,91E+00 | OTUD5; SLC22A6; HSPB6; NR2E1; PIGW; TOB2; GLI3; ACTG1; MECOM; PPME1; BSG; NDST3; ZKSCAN2; ZSWIM5; SOX5; SCARA3; DONSON; DDX10; YOD1; SORBS2; MSRA; RAD51; STIM1; HOXB4; SMCR8; SF1 |
| XBP1 (human) | 4,19E-02 | 8,44E-01 | 8,64E-03 | 9,09E-02 | -1,78E+00 | 5,65E+00 | FARP1; HSPB6; SLC20A1; EMILIN2; GLI3; SSH1 |
| CEBPB (human) | 3,50E-02 | 8,44E-01 | 2,73E-03 | 5,72E-02 | -1,65E+00 | 5,55E+00 | PIGS; RPIA; TMED9; HSPB6; NDST3; SPRY3; RFFL; CBL; SLC25A4; ETS2; GLI3 |
| AHR (mouse) | 4,23E-02 | 8,44E-01 | 1,08E-02 | 1,03E-01 | -1,75E+00 | 5,52E+00 | ERBB3; AI464131; DDX10; TNFAIP8L2; SERF2 |
| WT1 (human) | 2,87E-02 | 8,44E-01 | 6,23E-04 | 3,01E-02 | -1,54E+00 | 5,48E+00 | GTF3C2; OTUD5; SCARA3; NLK; SLC2A6; GLI3; ACTG1; SSH1; ADCY9; TMEM214; STIM1; PQLC2; NDST3; SPRY2; SLC25A23; SERF2; SF1; SOX5 |
| ELK4 (human) | 4,03E-02 | 8,44E-01 | 3,25E-03 | 5,72E-02 | -1,67E+00 | 5,36E+00 | RAD51; TMEM214; MECOM; HSPB6; CAT; RHO; YOD1; SMCR8; SORBS2; SSH1; SOX5 |
| JUN (human) | 3,79E-02 | 8,44E-01 | 7,61E-04 | 3,07E-02 | -1,53E+00 | 5,01E+00 | PIGS; HSPB6; SLC20A1; NOTCH4; YOD1; SLC2A6; ETS2; GLI3; SSH1; RPIA; PQLC2; CYP1A2; SPRY3; EMILIN2; SLC25A23; ZSWIM5; SLC25A4; SERF2; SF1 |
| STAT1 (human) | 4,53E-02 | 8,44E-01 | 3,17E-03 | 5,72E-02 | -1,57E+00 | 4,85E+00 | RPIA; FARP1; TMED9; RAD51; PPME1; BSG; SPRY3; ELP2; NLK; CBL; SNX5; SOX5 |
| ZFHX3 (human) | 4,96E-02 | 8,54E-01 | 4,21E-03 | 6,79E-02 | -1,61E+00 | 4,83E+00 | GTF3C2; RAD51; STIM1; BSG; CYP1A2; DONSON; EIF4EBP2; DDX10; DNHD1; YOD1; SORBS2 |

**Table S7. Enriched KEGG and REACTOME pathways for the genes targeted by miRNAs differentially expressed between 12m and 12mCR animals.**

| **Term** | **P-value** | **Adjusted P-value** | **Old P-value** | **Old Adjusted P value** | **Z score** | **Combined Score** | **Genes** |
| --- | --- | --- | --- | --- | --- | --- | --- |
| **KEGG Pathways** |  |  |  |  |  |  |  |
| Neurotrophin signaling pathway_hsa04722 | 6,56E-03 | 3,29E-01 | 1,42E-02 | 6,30E-01 | -1,83E+00 | 9,21E+00 | RPS6KA3; RPS6KA5; MAPK7; PRDM4; PIK3R2; SH2B3 |
| Carbon metabolism_hsa01200 | 4,91E-03 | 3,29E-01 | 1,09E-02 | 6,30E-01 | -1,69E+00 | 8,96E+00 | RPIA; MDH1; GPT2; SHMT1; CAT; PDHB |
| Regulation of lipolysis in adipocytes_hsa04923 | 7,52E-03 | 3,29E-01 | 1,44E-02 | 6,30E-01 | -1,79E+00 | 8,76E+00 | GNAI3; PRKG2; PIK3R2; TSHB |
| Endocytosis _hsa04144 | 1,03E-02 | 3,63E-01 | 2,48E-02 | 6,80E-01 | -1,84E+00 | 8,41E+00 | ACAP3; TGFB1; RAB31; GRK5; ERBB3; ZFYVE20; WASL; SNX5; EPN1 |
| Glycosylphosphatidylinositol(GPI)-anchor biosynthesis_hsa00563 | 4,82E-03 | 3,29E-01 | 9,32E-03 | 6,30E-01 | -1,57E+00 | 8,38E+00 | PIGS; PIGN; PIGW |
| Inflammatory bowel disease (IBD)_hsa05321 | 1,26E-02 | 3,63E-01 | 2,29E-02 | 6,80E-01 | -1,74E+00 | 7,62E+00 | IL1A; TGFB1; IFNGR1; IL21R |
| Renal cell carcinoma_hsa05211 | 1,33E-02 | 3,63E-01 | 2,40E-02 | 6,80E-01 | -1,67E+00 | 7,21E+00 | ARNT2; TGFB1; PAK7; PIK3R2 |
| Glyoxylate and dicarboxylate metabolism_hsa00630 | 6,66E-03 | 3,29E-01 | 1,24E-02 | 6,30E-01 | -1,39E+00 | 6,95E+00 | MDH1; SHMT1; CAT |
| Chronic myeloid leukemia_hsa05220 | 1,86E-02 | 4,26E-01 | 3,26E-02 | 7,36E-01 | -1,68E+00 | 6,68E+00 | TGFB1; MECOM; CTBP1; PIK3R2 |
| Oxytocin signaling pathway_hsa04921 | 2,29E-02 | 4,57E-01 | 4,41E-02 | 7,36E-01 | -1,71E+00 | 6,45E+00 | MAPK7; GNAI3; PIK3R2; OXT; CAMKK1; ACTG1 |
| **MAPK signaling pathway_hsa04010** | 2,62E-02 | 4,64E-01 | 5,47E-02 | 7,36E-01 | -1,72E+00 | 6,26E+00 | IL1A; RPS6KA3; TGFB1; RPS6KA5; MAPK7; MECOM; DUSP1; NLK |
| Adherens junction_hsa04520 | 1,94E-02 | 4,26E-01 | 3,40E-02 | 7,36E-01 | -1,57E+00 | 6,18E+00 | CSNK2A1; WASL; NLK; ACTG1 |
| Platelet activation_hsa04611 | 2,75E-02 | 4,64E-01 | 4,94E-02 | 7,36E-01 | -1,60E+00 | 5,75E+00 | STIM1; GNAI3; PRKG2; PIK3R2; ACTG1 |
| Gap junction _hsa04540 | 3,40E-02 | 4,88E-01 | 5,65E-02 | 7,36E-01 | -1,39E+00 | 4,69E+00 | MAPK7; GNAI3; PRKG2; TUBB4A |
| Morphine addiction _hsa05032 | 3,77E-02 | 4,88E-01 | 6,21E-02 | 7,36E-01 | -1,43E+00 | 4,67E+00 | GRK5; GABRA4; GNAI3; PDE7A |
| FoxO signaling pathway _hsa04068 | 3,78E-02 | 4,88E-01 | 6,59E-02 | 7,36E-01 | -1,42E+00 | 4,65E+00 | TGFB1; CAT; PIK3R2; NLK; KLF2 |
| Salmonella infection _hsa05132 | 3,16E-02 | 4,88E-01 | 5,29E-02 | 7,36E-01 | -1,34E+00 | 4,62E+00 | IL1A; IFNGR1; WASL; ACTG1 |
| Herpes simplex infection _hsa05168 | 4,43E-02 | 5,11E-01 | 7,99E-02 | 7,36E-01 | -1,35E+00 | 4,20E+00 | UBE2R2; CSNK2A1; IFNGR1; MED8; TBPL1; SRSF7 |
| Pathogenic Escherichia coli infection_hsa05130 | 4,07E-02 | 4,95E-01 | 6,27E-02 | 7,36E-01 | -1,15E+00 | 3,68E+00 | WASL; TUBB4A; ACTG1 |
| **REACTOME Pathways** |  |  |  |  |  |  |  |
| Post-translational protein modification_R-HSA-597592 | 2,60E-03 | 3,03E-01 | 5,77E-03 | 6,26E-01 | -2,17E+00 | 1,29E+01 | PIGS; DPAGT1; PDIA3; PIGN; TRAPPC4; ST8SIA2; GOSR1; PIGW; MANEA; THBS1; ADAMTS15; HERC2; OS9; NUP98; TMED7; B4GALT4 |
| ERK/MAPK targets_R-HSA-198753 | 2,91E-03 | 3,03E-01 | 5,13E-03 | 6,26E-01 | -2,11E+00 | 1,23E+01 | RPS6KA3; RPS6KA5; MAPK7 |
| Metabolism of nitric oxide_R-HSA-202131 | 2,52E-03 | 3,03E-01 | 4,54E-03 | 6,26E-01 | -2,05E+00 | 1,23E+01 | PRKG2; ZDHHC21; WASL |
| eNOS activation and regulation _R-HSA-203765 | 2,52E-03 | 3,03E-01 | 4,54E-03 | 6,26E-01 | -2,02E+00 | 1,21E+01 | PRKG2; ZDHHC21; WASL |
| Metabolism of proteins _R-HSA-392499 | 4,14E-03 | 3,03E-01 | 1,02E-02 | 6,43E-01 | -2,10E+00 | 1,15E+01 | PIGS; DPAGT1; PIGN; RPL12; GOSR1; GNAI3; PIGW; MANEA; THBS1; ADAMTS15; HERC2; OS9; TMED7; TSHB; PDIA3; CSNK2A1; ST8SIA2; TRAPPC4; TUBB4A; ADRA2A; MSRA; SKIV2L; NUP98; MFGE8; SLC25A4; B4GALT4 |
| Syndecan interactions _R-HSA-3000170 | 2,52E-03 | 3,03E-01 | 4,54E-03 | 6,26E-01 | -1,92E+00 | 1,15E+01 | TGFB1; TRAPPC4; THBS1 |
| Hemostasis _R-HSA-109582 | 4,55E-03 | 3,03E-01 | 9,70E-03 | 6,43E-01 | -2,08E+00 | 1,12E+01 | TGFB1; ITGA1; GNAI3; PIK3R2; MIF; THBS1; ADRA2A; THBD; STIM1; KIFC2; PSAP; BSG; PRKG2; ESAM; DAGLB; SH2B3 |
| Nuclear Events (kinase and transcription factor activation) _R-HSA-198725 | 4,29E-03 | 3,03E-01 | 7,18E-03 | 6,26E-01 | -1,98E+00 | 1,08E+01 | RPS6KA3; RPS6KA5; MAPK7 |
| Post-translational modification: synthesis of GPI-anchored proteins _R-HSA-163125 | 4,82E-03 | 3,03E-01 | 7,96E-03 | 6,26E-01 | -1,96E+00 | 1,04E+01 | PIGS; PIGN; PIGW |
| MAPK targets/ Nuclear events mediated by MAP kinases_R-HSA-450282 | 8,09E-03 | 4,24E-01 | 1,26E-02 | 6,85E-01 | -2,09E+00 | 1,01E+01 | RPS6KA3; RPS6KA5; MAPK7 |
| Asparagine N-linked glycosylation_R-HSA-446203 | 1,03E-02 | 4,65E-01 | 1,80E-02 | 8,02E-01 | -2,02E+00 | 9,24E+00 | DPAGT1; PDIA3; OS9; ST8SIA2; TRAPPC4; GOSR1; TMED7; MANEA; B4GALT4 |
| Cell surface interactions at the vascular wall_R-HSA-202733 | 1,33E-02 | 5,56E-01 | 2,04E-02 | 8,02E-01 | -1,83E+00 | 7,91E+00 | THBD; BSG; ESAM; PIK3R2; MIF |
| Calnexin/calreticulin cycle _R-HSA-901042 | 1,78E-02 | 6,23E-01 | 2,56E-02 | 8,02E-01 | -1,84E+00 | 7,40E+00 | PDIA3; OS9 |
| Purine catabolism _R-HSA-74259 | 7,99E-03 | 4,24E-01 | 1,31E-02 | 6,85E-01 | -1,49E+00 | 7,20E+00 | NT5E; CAT |
| Non-integrin membrane-ECM interactions _R-HSA-3000171 | 2,03E-02 | 6,38E-01 | 2,87E-02 | 8,02E-01 | -1,82E+00 | 7,10E+00 | TGFB1; TRAPPC4; THBS1 |
| N-glycan trimming in the ER and Calnexin/Calreticulin cycle _R-HSA-532668 | 2,27E-02 | 6,79E-01 | 3,16E-02 | 8,02E-01 | -1,82E+00 | 6,90E+00 | PDIA3; OS9 |
| Transport to the Golgi and subsequent modification_R-HSA-948021 | 2,55E-02 | 6,95E-01 | 3,86E-02 | 8,02E-01 | -1,85E+00 | 6,80E+00 | TRAPPC4; ST8SIA2; GOSR1; TMED7; MANEA; B4GALT4 |
| Developmental Biology_R-HSA-1266738 | 4,71E-02 | 6,95E-01 | 8,46E-02 | 8,02E-01 | -2,13E+00 | 6,50E+00 | MYOG; TGFB1; CSNK2A1; KSR1; DUSP1; ST8SIA2; ITGA1; MED8; WASL; ACTG1; RPS6KA3; ABLIM2; NR6A1; RPS6KA5; ERBB3; PAK7; HOXB4 |
| Metabolism of nucleotides_R-HSA-15869 | 3,28E-02 | 6,95E-01 | 4,59E-02 | 8,02E-01 | -1,83E+00 | 6,26E+00 | DUT; NT5E; CAT; AK1 |
| Interleukin-7 signaling_R-HSA-1266695 | 9,68E-03 | 4,65E-01 | 1,53E-02 | 7,40E-01 | -1,33E+00 | 6,16E+00 | IL7; PIK3R2 |
| Pyruvate metabolism and Citric Acid (TCA) cycle_R-HSA-71406 | 2,88E-02 | 6,95E-01 | 3,96E-02 | 8,02E-01 | -1,63E+00 | 5,77E+00 | ADHFE1; BSG; PDHB |
| Synthesis of glycosylphosphatidylinositol (GPI)_R-HSA-162710 | 2,02E-02 | 6,38E-01 | 2,86E-02 | 8,02E-01 | -1,48E+00 | 5,77E+00 | PIGN; PIGW |
| G alpha (s) signalling events_R-HSA-418555 | 4,78E-02 | 6,95E-01 | 6,72E-02 | 8,02E-01 | -1,85E+00 | 5,63E+00 | GRK5; GNAI3; PDE7A; CRHR1; TSHB |
| Chaperonin-mediated protein folding_R-HSA-390466 | 4,31E-02 | 6,95E-01 | 5,92E-02 | 8,02E-01 | -1,78E+00 | 5,60E+00 | CSNK2A1; GNAI3; SKIV2L; TUBB4A |
| DCC mediated attractive signaling_R-HSA-418885 | 1,56E-02 | 6,13E-01 | 2,28E-02 | 8,02E-01 | -1,33E+00 | 5,52E+00 | ABLIM2; WASL |
| N-Glycan antennae elongation_R-HSA-975577 | 1,78E-02 | 6,23E-01 | 2,56E-02 | 8,02E-01 | -1,36E+00 | 5,48E+00 | ST8SIA2; B4GALT4 |
| CREB phosphorylation_R-HSA-199920 | 3,83E-03 | 3,03E-01 | 7,33E-03 | 6,26E-01 | -9,44E-01 | 5,25E+00 | RPS6KA3; RPS6KA5 |
| L1CAM interactions_R-HSA-373760 | 4,45E-02 | 6,95E-01 | 6,10E-02 | 8,02E-01 | -1,65E+00 | 5,13E+00 | RPS6KA3; RPS6KA5; CSNK2A1; ITGA1 |
| Amino acid synthesis and interconversion (transamination)_R-HSA-70614 | 4,65E-02 | 6,95E-01 | 6,01E-02 | 8,02E-01 | -1,66E+00 | 5,08E+00 | OAT; GPT2 |
| Nephrin interactions_R-HSA-373753 | 3,68E-02 | 6,95E-01 | 4,87E-02 | 8,02E-01 | -1,54E+00 | 5,07E+00 | PIK3R2; WASL |
| Pyrimidine metabolism_R-HSA-73848 | 4,32E-02 | 6,95E-01 | 5,62E-02 | 8,02E-01 | -1,47E+00 | 4,61E+00 | DUT; NT5E |
| N-glycan antennae elongation in the medial/trans-Golgi_R-HSA-975576 | 4,99E-02 | 6,95E-01 | 6,42E-02 | 8,02E-01 | -1,52E+00 | 4,56E+00 | ST8SIA2; B4GALT4 |
| Recycling pathway of L1_R-HSA-437239 | 4,99E-02 | 6,95E-01 | 6,42E-02 | 8,02E-01 | -1,47E+00 | 4,40E+00 | RPS6KA3; RPS6KA5 |

**Table S8. Enriched gene ontology (GO) terms for molecular function and biological process for the genes targeted by miRNAs differentially expressed between 12m vs 12mCR animals.**

| **Term** | **P-value** | **Adjusted P-value** | **Old P-value** | **Old Adjusted P-value** | **Z score** | **Combined Score** | **Genes** |
| --- | --- | --- | --- | --- | --- | --- | --- |
| **GO Terms Molecular function** |  |  |  |  |  |  |  |
| protein homodimerization activity (GO:0042803) | 1,66E-02 | 3,34E-01 | 3,30E-02 | 3,82E-01 | -6,42E+00 | 2,63E+01 | CARS; HSPB6; SHMT1; ABAT; FAM109A; ADRA2A; NR6A1; ERBB3; ALS2; BHLHE40; PSAP; CAT; HMOX1; MAP3K10; TRIM37 |
| protein serine/threonine kinase activity (GO:0004674) | 8,51E-03 | 3,25E-01 | 1,60E-02 | 3,82E-01 | -4,92E+00 | 2,34E+01 | RPS6KA3; SNRK; RPS6KA5; MAPK7; CSNK2A1; NEK4; GRK5; MAP3K10; TLK1; NLK |
| pyridoxal phosphate binding (GO:0030170) | 2,91E-03 | 2,28E-01 | 5,19E-03 | 3,82E-01 | -3,30E+00 | 1,92E+01 | OAT; SHMT1; ABAT |
| ATP transmembrane transporter activity (GO:0005347) | 2,76E-03 | 2,28E-01 | 5,80E-03 | 3,82E-01 | -2,52E+00 | 1,49E+01 | SLC25A23; SLC25A25 |
| ADP transmembrane transporter activity (GO:0015217) | 2,76E-03 | 2,28E-01 | 5,80E-03 | 3,82E-01 | -2,52E+00 | 1,49E+01 | SLC25A23; SLC25A25 |
| 2-acylglycerol-3-phosphate O-acyltransferase activity (GO:0047144) | 6,45E-03 | 3,25E-01 | 1,11E-02 | 3,82E-01 | -2,61E+00 | 1,32E+01 | MBOAT7; MBOAT2 |
| 1-acylglycerol-3-phosphate O-acyltransferase activity (GO:0003841) | 2,53E-02 | 3,34E-01 | 3,50E-02 | 3,82E-01 | -3,44E+00 | 1,27E+01 | MBOAT7; MBOAT2 |
| protein kinase activity (GO:0004672) | 3,70E-02 | 3,34E-01 | 5,54E-02 | 3,82E-01 | -3,75E+00 | 1,23E+01 | RPS6KA3; RPS6KA5; KSR1; MAP3K10; PRKG2; NLK |
| proteoglycan binding (GO:0043394) | 7,99E-03 | 3,25E-01 | 1,31E-02 | 3,82E-01 | -2,52E+00 | 1,22E+01 | THBS1; CTSB |
| Rac guanyl-nucleotide exchange factor activity (GO:0030676) | 1,15E-02 | 3,34E-01 | 1,78E-02 | 3,82E-01 | -2,67E+00 | 1,19E+01 | FARP1; ALS2 |
| bHLH transcription factor binding (GO:0043425) | 3,08E-02 | 3,34E-01 | 4,18E-02 | 3,82E-01 | -3,02E+00 | 1,05E+01 | BHLHE40; MAP3K10 |
| Rab guanyl-nucleotide exchange factor activity (GO:0017112) | 3,19E-02 | 3,34E-01 | 4,40E-02 | 3,82E-01 | -2,98E+00 | 1,03E+01 | ALS2; TRAPPC4; SMCR8 |
| MAP kinase activity (GO:0004707) | 9,68E-03 | 3,25E-01 | 1,54E-02 | 3,82E-01 | -2,13E+00 | 9,88E+00 | MAPK7; NLK |
| glycoprotein binding (GO:0001948) | 1,90E-02 | 3,34E-01 | 2,74E-02 | 3,82E-01 | -2,45E+00 | 9,71E+00 | TGFB1; OS9; THBS1 |
| protein phosphatase binding (GO:0019903) | 3,19E-02 | 3,34E-01 | 4,40E-02 | 3,82E-01 | -2,76E+00 | 9,50E+00 | PPME1; ITGA1; PIK3R2 |
| GDP binding (GO:0019003) | 3,03E-02 | 3,34E-01 | 4,20E-02 | 3,82E-01 | -2,71E+00 | 9,49E+00 | RAB31; GNAI3; ARL8B |
| heme binding (GO:0020037) | 4,45E-02 | 3,34E-01 | 5,97E-02 | 3,82E-01 | -2,73E+00 | 8,49E+00 | CYP1A2; CAT; HMOX1 |
| organic anion transmembrane transporter activity (GO:0008514) | 3,38E-02 | 3,34E-01 | 4,53E-02 | 3,82E-01 | -2,13E+00 | 7,23E+00 | SLC22A6; ABCC5 |
| phosphatidylserine binding (GO:0001786) | 4,65E-02 | 3,34E-01 | 6,05E-02 | 3,82E-01 | -2,18E+00 | 6,69E+00 | JPH2; THBS1 |
| **GO Terms Biological Process** |  |  |  |  |  |  |  |
| negative regulation of gene expression (GO:0010629) | 2,31E-03 | 3,24E-01 | 3,82E-03 | 3,96E-01 | -3,03E+00 | 1,84E+01 | DLL4; TRIM6; TGFB1; NDFIP1; SMCR8; MIF |
| protein phosphorylation (GO:0006468) | 1,13E-02 | 3,55E-01 | 1,79E-02 | 3,96E-01 | -3,97E+00 | 1,78E+01 | SNRK; TGFB1; RPS6KA5; CSNK2A1; NEK4; KSR1; CTBP1; PRKG2; TLK1; NLK |
| dorsal aorta morphogenesis (GO:0035912) | 3,83E-03 | 3,24E-01 | 7,02E-03 | 3,96E-01 | -2,89E+00 | 1,61E+01 | DLL4; HEY1 |
| positive regulation of fibroblast migration (GO:0010763) | 2,76E-03 | 3,24E-01 | 5,51E-03 | 3,96E-01 | -2,69E+00 | 1,59E+01 | TGFB1; THBS1 |
| cardiac ventricle morphogenesis (GO:0003208) | 3,83E-03 | 3,24E-01 | 7,02E-03 | 3,96E-01 | -2,70E+00 | 1,50E+01 | DLL4; HEY1 |
| negative regulation of transcription, DNA-templated (GO:0045892) | 2,75E-02 | 3,55E-01 | 4,16E-02 | 3,96E-01 | -4,07E+00 | 1,46E+01 | MAGEL2; DAP; TGFB1; RPS6KA5; HEY1; CTBP1; BHLHE40; ZKSCAN3; MAP3K10; EPC1; ARID5B |
| negative regulation of transcription from RNA polymerase II promoter (GO:0000122) | 3,28E-02 | 3,55E-01 | 4,98E-02 | 3,96E-01 | -4,10E+00 | 1,40E+01 | DLL4; ZFP36; NR6A1; HEY1; CTBP1; BHLHE40; ZKSCAN3; EPC1; ARID5B; TRIM37; RREB1; ETS2 |
| positive regulation of transcription from RNA polymerase II promoter in response to stress (GO:0036003) | 2,76E-03 | 3,24E-01 | 5,51E-03 | 3,96E-01 | -2,26E+00 | 1,33E+01 | MAPK7; KLF2 |
| regulation of nitric-oxide synthase activity (GO:0050999) | 3,33E-03 | 3,24E-01 | 5,43E-03 | 3,96E-01 | -2,27E+00 | 1,29E+01 | PRKG2; ZDHHC21; WASL |
| UV protection (GO:0009650) | 7,99E-03 | 3,55E-01 | 1,25E-02 | 3,96E-01 | -2,60E+00 | 1,26E+01 | SCARA3; CAT |
| cellular response to heat (GO:0034605) | 5,40E-03 | 3,51E-01 | 8,28E-03 | 3,96E-01 | -2,40E+00 | 1,25E+01 | IL1A; HMOX1; THBS1 |
| Notch signaling involved in heart development (GO:0061314) | 6,45E-03 | 3,51E-01 | 1,05E-02 | 3,96E-01 | -2,43E+00 | 1,23E+01 | DLL4; HEY1 |
| protein retention in ER lumen (GO:0006621) | 1,86E-03 | 3,24E-01 | 4,17E-03 | 3,96E-01 | -1,75E+00 | 1,10E+01 | PDIA3; OS9 |
| peptidyl-threonine phosphorylation (GO:0018107) | 1,01E-02 | 3,55E-01 | 1,45E-02 | 3,96E-01 | -2,38E+00 | 1,09E+01 | CSNK2A1; MAP3K10; NLK; CAMKK1 |
| negative regulation of fibrinolysis (GO:0051918) | 6,45E-03 | 3,51E-01 | 1,05E-02 | 3,96E-01 | -2,16E+00 | 1,09E+01 | THBD; THBS1 |
| cellular metal ion homeostasis (GO:0006875) | 2,76E-03 | 3,24E-01 | 5,51E-03 | 3,96E-01 | -1,78E+00 | 1,05E+01 | TESMIN; LETMD1 |
| calcium ion transport into cytosol (GO:0060402) | 1,15E-02 | 3,55E-01 | 1,69E-02 | 3,96E-01 | -2,26E+00 | 1,01E+01 | JPH4; JPH2 |
| response to endoplasmic reticulum stress (GO:0034976) | 1,01E-02 | 3,55E-01 | 1,45E-02 | 3,96E-01 | -2,19E+00 | 1,00E+01 | PDIA3; OS9; PIK3R2; THBS1 |
| retrograde transport, endosome to Golgi (GO:0042147) | 1,62E-02 | 3,55E-01 | 2,23E-02 | 3,96E-01 | -2,18E+00 | 8,99E+00 | MAGEL2; GOSR1; FAM109A; SNX5 |
| adenylate cyclase-inhibiting G-protein coupled receptor signaling pathway (GO:0007193) | 8,87E-03 | 3,55E-01 | 1,29E-02 | 3,96E-01 | -1,90E+00 | 8,98E+00 | PSAP; GNAI3; GPR37L1 |
| response to progesterone (GO:0032570) | 1,56E-02 | 3,55E-01 | 2,19E-02 | 3,96E-01 | -1,97E+00 | 8,19E+00 | TGFB1; THBS1 |
| intracellular signal transduction (GO:0035556) | 4,84E-02 | 3,55E-01 | 6,58E-02 | 3,96E-01 | -2,64E+00 | 8,00E+00 | RPS6KA3; SNRK; RPS6KA5; HMOX1; TLK1; NLK; CAMKK1 |
| bone resorption (GO:0045453) | 5,06E-03 | 3,51E-01 | 8,69E-03 | 3,96E-01 | -1,45E+00 | 7,66E+00 | IL7; RAB3D |
| regulation of ryanodine-sensitive calcium-release channel activity (GO:0060314) | 1,56E-02 | 3,55E-01 | 2,19E-02 | 3,96E-01 | -1,61E+00 | 6,71E+00 | JPH4; JPH2 |
| positive regulation of transcription regulatory region DNA binding (GO:2000679) | 1,35E-02 | 3,55E-01 | 1,93E-02 | 3,96E-01 | -1,48E+00 | 6,36E+00 | TRIM6; TGFB1 |
| negative regulation of cysteine-type endopeptidase activity involved in apoptotic process (GO:0043154) | 2,43E-02 | 3,55E-01 | 3,21E-02 | 3,96E-01 | -1,70E+00 | 6,31E+00 | RPS6KA3; CSNK2A1; THBS1 |
| regulation of autophagy (GO:0010506) | 2,16E-02 | 3,55E-01 | 2,88E-02 | 3,96E-01 | -1,63E+00 | 6,27E+00 | PSAP; SMCR8; PIK3R2 |
| positive regulation of protein metabolic process (GO:0051247) | 7,99E-03 | 3,55E-01 | 1,25E-02 | 3,96E-01 | -1,23E+00 | 5,96E+00 | MAPK7; KLF2 |
| histone H2A acetylation (GO:0043968) | 2,02E-02 | 3,55E-01 | 2,74E-02 | 3,96E-01 | -1,44E+00 | 5,63E+00 | EPC1; YEATS4 |
| negative regulation of Notch signaling pathway (GO:0045746) | 2,27E-02 | 3,55E-01 | 3,03E-02 | 3,96E-01 | -1,34E+00 | 5,06E+00 | DLL4; HEY1 |
| negative regulation of inclusion body assembly (GO:0090084) | 6,45E-03 | 3,51E-01 | 1,05E-02 | 3,96E-01 | -9,27E-01 | 4,68E+00 | DNAJA4; SACS |
| preassembly of GPI anchor in ER membrane (GO:0016254) | 1,78E-02 | 3,55E-01 | 2,46E-02 | 3,96E-01 | -1,09E+00 | 4,40E+00 | PIGN; PIGW |
| positive regulation of cytokine secretion (GO:0050715) | 2,80E-02 | 3,55E-01 | 3,66E-02 | 3,96E-01 | -1,18E+00 | 4,21E+00 | IL1A; MIF |
| 2-oxoglutarate metabolic process (GO:0006103) | 1,56E-02 | 3,55E-01 | 2,19E-02 | 3,96E-01 | -9,68E-01 | 4,03E+00 | GPT2; ADHFE1 |
| Ras protein signal transduction (GO:0007265) | 4,26E-02 | 3,55E-01 | 5,38E-02 | 3,96E-01 | -1,25E+00 | 3,96E+00 | KSR1; RREB1; ADRA2A |
| cellular response to laminar fluid shear stress (GO:0071499) | 3,83E-03 | 3,24E-01 | 7,02E-03 | 3,96E-01 | -6,87E-01 | 3,82E+00 | MAPK7; KLF2 |
| positive regulation of peptidyl-serine phosphorylation (GO:0033138) | 4,45E-02 | 3,55E-01 | 5,61E-02 | 3,96E-01 | -1,19E+00 | 3,70E+00 | TRIM6; TGFB1; MIF |
| positive regulation of chemotaxis (GO:0050921) | 1,15E-02 | 3,55E-01 | 1,69E-02 | 3,96E-01 | -7,22E-01 | 3,22E+00 | TGFB1; THBS1 |
| negative regulation of blood vessel endothelial cell migration (GO:0043537) | 1,56E-02 | 3,55E-01 | 2,19E-02 | 3,96E-01 | -7,45E-01 | 3,10E+00 | TGFB1; THBS1 |
| negative regulation of adenylate cyclase activity (GO:0007194) | 3,38E-02 | 3,55E-01 | 4,32E-02 | 3,96E-01 | -8,33E-01 | 2,82E+00 | GNAI3; ADRA2A |
| cellular response to fibroblast growth factor stimulus (GO:0044344) | 2,53E-02 | 3,55E-01 | 3,34E-02 | 3,96E-01 | -7,44E-01 | 2,74E+00 | DLL4; ZFP36 |
| negative regulation of ERBB signaling pathway (GO:1901185) | 1,35E-02 | 3,55E-01 | 1,93E-02 | 3,96E-01 | -6,21E-01 | 2,67E+00 | PTPN18; ERBB3 |
| phosphate-containing compound metabolic process (GO:0006796) | 2,02E-02 | 3,55E-01 | 2,74E-02 | 3,96E-01 | -6,76E-01 | 2,64E+00 | TGFB1; SLC20A1 |
| positive regulation of blood vessel endothelial cell migration (GO:0043536) | 2,80E-02 | 3,55E-01 | 3,66E-02 | 3,96E-01 | -6,32E-01 | 2,26E+00 | TGFB1; THBS1 |
| positive regulation of B cell proliferation (GO:0030890) | 3,99E-02 | 3,55E-01 | 5,03E-02 | 3,96E-01 | -5,04E-01 | 1,62E+00 | IL7; MIF |
| response to hydrogen peroxide (GO:0042542) | 2,02E-02 | 3,55E-01 | 2,74E-02 | 3,96E-01 | -3,42E-01 | 1,34E+00 | CAT; HMOX1 |
| positive regulation of vascular endothelial growth factor production (GO:0010575) | 4,32E-02 | 3,55E-01 | 5,40E-02 | 3,96E-01 | -3,40E-01 | 1,07E+00 | IL1A; TGFB1 |
| negative regulation of endothelial cell migration (GO:0010596) | 2,27E-02 | 3,55E-01 | 3,03E-02 | 3,96E-01 | -2,45E-01 | 9,29E-01 | DLL4; THBS1 |
| positive regulation of phosphorylation (GO:0042327) | 2,80E-02 | 3,55E-01 | 3,66E-02 | 3,96E-01 | -2,54E-01 | 9,08E-01 | MIF; THBS1 |
| positive regulation of peptidyl-threonine phosphorylation (GO:0010800) | 3,38E-02 | 3,55E-01 | 4,32E-02 | 3,96E-01 | -2,14E-01 | 7,26E-01 | TRIM6; TGFB1 |
| cellular amino acid biosynthetic process (GO:0008652) | 4,65E-02 | 3,55E-01 | 5,78E-02 | 3,96E-01 | -2,20E-01 | 6,74E-01 | OAT; GPT2 |
| protein tetramerization (GO:0051262) | 3,68E-02 | 3,55E-01 | 4,67E-02 | 3,96E-01 | -1,11E-01 | 3,66E-01 | SHMT1; CAT |
| **positive regulation of phosphatidylinositol 3-kinase activity (GO:0043552)** | 4,32E-02 | 3,55E-01 | 5,40E-02 | 3,96E-01 | -5,41E-02 | 1,70E-01 | TGFB1; TNFAIP8L3 |
| pyruvate metabolic process (GO:0006090) | 2,80E-02 | 3,55E-01 | 3,66E-02 | 3,96E-01 | -4,25E-02 | 1,52E-01 | BSG; PDHB |
| positive regulation of smooth muscle cell proliferation (GO:0048661) | 4,32E-02 | 3,55E-01 | 5,40E-02 | 3,96E-01 | -4,37E-02 | 1,37E-01 | HMOX1; THBS1 |
| cAMP-mediated signaling (GO:0019933) | 3,68E-02 | 3,55E-01 | 4,67E-02 | 3,96E-01 | -3,74E-02 | 1,23E-01 | MAPK7; KSR1 |
| negative regulation of extrinsic apoptotic signaling pathway in absence of ligand (GO:2001240) | 3,68E-02 | 3,55E-01 | 4,67E-02 | 3,96E-01 | -2,58E-02 | 8,51E-02 | IL1A; MAPK7 |
| tricarboxylic acid cycle (GO:0006099) | 4,99E-02 | 3,55E-01 | 6,16E-02 | 3,96E-01 | -1,51E-02 | 4,52E-02 | MDH1; PDHB |
| **phosphatidylinositol 3-kinase signaling (GO:0014065)** | 4,99E-02 | 3,55E-01 | 6,16E-02 | 3,96E-01 | -1,15E-02 | 3,44E-02 | ERBB3; PIK3R2 |
| peptidyl-tyrosine dephosphorylation (GO:0035335) | 2,80E-02 | 3,55E-01 | 3,66E-02 | 3,96E-01 | -1,90E-03 | 6,79E-03 | PTPRZ1; DUSP1 |
| mitotic cell cycle checkpoint (GO:0007093) | 4,65E-02 | 3,55E-01 | 5,78E-02 | 3,96E-01 | 1,92E-01 | -5,90E-01 | TGFB1; NABP1 |
| vesicle organization (GO:0016050) | 2,80E-02 | 3,55E-01 | 3,66E-02 | 3,96E-01 | 1,72E-01 | -6,15E-01 | WASL; SNX5 |

**Table S9. Enriched TRANSFAC and JASPAR terms for the transcription factor genes targeted by miRNAs differentially expressed between 12m vs 12mCR animals.**

| **Term** | **P-value** | **Adjusted P-value** | **Old P-value** | **Old Adjusted P value** | **Z score** | **Combined Score** | **Genes** |
| --- | --- | --- | --- | --- | --- | --- | --- |
| SP1 (mouse) | 1,34E-04 | 3,63E-02 | 1,15E-09 | 3,12E-07 | -1,64E+00 | 1,46E+01 | COX4I1; RAB3D; ACTG1; RAB43; RPS6KA3; RPS6KA5; UBASH3B; HEY1; TLK1; TMED7; MYOG; DUSP1; ARHGEF17; COASY; SAP30; ACAP3; 0610010F05RIK; STIM1; UBE2R2; KIFC2; TOX; RTN4R; CBFB; CTBP1; SLC20A1; TOB2; CRHR1; DLL4; MAPK7; RFFL; ZSWIM5; SH2B3; RREB1; SERF2; FARP1; DUT; TGFB1; MBD6; EPB4,1; ZFP318; MBOAT7; PLCL2; MBOAT2; HELZ; MPP5; KLF2; SSH1; SNRK; PPFIBP1; DNAJA4; BHLHE40; PQLC2; PDE7A; GRINA |
| XBP1 (human) | 2,03E-03 | 2,61E-01 | 2,50E-05 | 9,67E-04 | -1,89E+00 | 1,17E+01 | FARP1; TGFB1; HSPB6; SLC20A1; YTHDC1; SHMT1; SSH1; SAP30; FAM155A; TSGA10; PPFIBP1; RPS6KA5; DNAJA4; IL21R; PGGT1B; EMILIN2; TMED7; DLGAP2 |
| TP53 (mouse) | 2,89E-03 | 2,61E-01 | 4,53E-06 | 3,07E-04 | -1,72E+00 | 1,00E+01 | DPAGT1; OAT; DDX46; YTHDC1; SHMT1; AKAP17B; LMBR1; BSG; EIF4EBP2; RFFL; ABCB1B; SLC35A5; SNX5; SERF2; SLC25A25; IKBIP; KSR1; TRAPPC4; GPT2; SMARCA5; DDX10; 0610010F05RIK; STIM1; GPD1; CYP1A2; IGDCC3; NAA16; MCM2; SF1; GRINA |
| PCBP1 (human) | 7,90E-03 | 4,35E-01 | 1,74E-05 | 7,88E-04 | -1,72E+00 | 8,34E+00 | CTBP1; KCNA2; ACTG1; DLL4; RAB43; LMBR1; PPP2R4; HEY1; BSG; DIP2C; SH2B3; RREB1; FARP1; DUSP1; ARHGEF17; MBOAT7; GPT2; SEMA4C; HELZ; EPN1; SSH1; ACAP3; KIFC2; STIM1; DNAJA4; BHLHE40; MAP3K10; TOX; B4GALT4; SF1 |
| ZNF281 (human) | 1,04E-02 | 4,72E-01 | 7,84E-05 | 1,93E-03 | -1,78E+00 | 8,11E+00 | MPP1; MBD6; HSPB6; CBFB; RPL12; MBOAT2; ACTG1; SSH1; DLL4; UNC80; ACAP3; TRIM6; LMBR1; PCSK1N; HEY1; BSG; HOXB4; PAK7; ZKSCAN2; TOX; SH2B3; RREB1; GPR125 |
| RELA (mouse) | 8,03E-03 | 4,35E-01 | 8,11E-06 | 4,39E-04 | -1,61E+00 | 7,78E+00 | RTN4R; COX4I1; MAK16; AK1; GOSR1; AKAP17B; NR2E1; PIGW; JPH4; LMBR1; ABLIM2; MAPK7; ATCAY; ZMYM3; PPP2R4; EME2; RHO; EMILIN2; SKP1A; DLGAP2; SLC25A25; CAR2; HSDL2; KSR1; BLCAP; NR6A1; ALS2; STIM1; FAM131A; MAP3K10; ESAM; NUP98; TOX; NSMAF; MCM2 |
| Tal1::Gata1 (mouse) | 2,53E-02 | 7,28E-01 | 1,47E-02 | 6,42E-02 | -2,07E+00 | 7,61E+00 | ARNT2; GRK5 |
| PLAU (human) | 1,40E-02 | 5,44E-01 | 4,53E-05 | 1,54E-03 | -1,71E+00 | 7,31E+00 | YTHDC1; SHMT1; NR2E1; LMBR1; IL21R; EMILIN2; DIP2C; TMED7; SNX5; SLC25A25; FARP1; DUSP1; TRAPPC4; ADHFE1; ST8SIA2; SEMA4C; DDX10; ELP2; SSH1; PPFIBP1; DAP; NR6A1; ALS2; FAM131A; NUP98; PDE7A; SLC25A14; SF1; MCM2 |
| EGR1 (mouse) | 2,60E-02 | 7,28E-01 | 7,05E-05 | 1,91E-03 | -1,60E+00 | 5,86E+00 | CTBP1; RAB3D; ZDHHC21; ACTG1; RPS6KA3; UBASH3B; AKAP14; PSAP; TLK1; TNFAIP8L3; SLC25A23; SLC35A5; SLC25A25; ARNT2; COL27A1; EPB4,1; ZFP318; GABRA4; RHBDF2; PLCL2; SMARCA5; MBOAT2; ADRA2A; KLF2; ACAP3; DAP; 0610010F05RIK; UBE2R2; STIM1; ZFP395; SLC25A14; MCM2 |
| TCFAP2A (human) | 4,00E-02 | 9,85E-01 | 2,76E-04 | 4,98E-03 | -1,64E+00 | 5,27E+00 | CBFB; CTBP1; ATL1; KCNA2; GREB1L; ACTG1; HEY1; GRK5; DIP2C; SLC25A23; RREB1; CTSB; FARP1; RPS6KL1; ARHGEF17; HELZ; SSH1; FAM155A; ACAP3; KIFC2; STIM1; DNAJA4; BHLHE40; MAP3K10; PDE7A; GPR125; SF1 |
| E2F1 (human) | 2,69E-02 | 7,28E-01 | 2,19E-07 | 2,97E-05 | -1,45E+00 | 5,26E+00 | OTUD5; HSPB6; PIGN; NR2E1; PIGW; ACTG1; THBD; LMBR1; RPS6KA5; HERC2; MECOM; HEY1; PPME1; POGZ; BSG; NDST3; EPC1; DIP2C; SLC35A5; PDIA3; SCARA3; ATP6V1G2; DUSP1; SLC6A15; LMO3; MED8; ARID5B; DDX10; MIF; ACAP3; ALS2; STIM1; KIFC2; HOXB4; SMCR8; MFGE8; TOX; SLC25A14; B4GALT4; SF1; SLC22A6; MAGEL2; CTBP1; MAK16; GNAI3; TOB2; DLL4; NT5E; MAPK7; ABLIM2; GRK5; ZKSCAN2; ZSWIM5; RREB1; ARNT2; DUT; NEK4; TRAPPC4; MBOAT7; ST8SIA2; SEMA4C; PLCL2; DONSON; MBOAT2; HELZ; MSRA; TSGA10; SNX18; DNAJA4; BHLHE40; TRIM37; NAA16 |

**Table S10. Screening of 19 differentially expressed matching miRNAs between comparisons in 3m *vs* 12m and 12m *vs* 12mCR.**

| **KEGG pathways** | **p-value** | **miRNAs** | **Genes** |
| --- | --- | --- | --- |
| Focal adhesion | 4,37E+05 | 17 | Sos2, Pipk5k1c, **Pik3r1**, Ibsp, Pdpk1, Pi3kr3, Col2a1, Gsk3b, Pdgfrb, Kdr, Rap1a, Col3a1, Map2k1, Col6a6, Thbs1, Col6a3, Reln, Rock1, Igf1, Col1a1, Pak1, Col27a1, Itga5, Itga11, Diap1, Vegfa, Col4a4, Itga, Mapk3, Braf, Mapk8, Rac1, Mylk3, Lamc3, Igf1r, Rock2, Sos1, Arhgap5, Bcl2, Col5a2, Col4a6, Col24a6, Col24a1, Col5a3, Pdgfb, Col1a2, Vcl, Ppp1cb, Flna, Actg1, Elk1, Lamc1, Col4a1, Col11a1, Cav1, Itga7, **Pten**, Col6a2, Itgb8, Mylk4, Pak2, Col4a2, Col5a1, Pdgfa, Cav2, Mapk8, Itga9, Pak4 |
| PI3K-Akt signaling pathway | 4,37E+05 | 18 | Prkaa2, Tsc1, Sos2, Fgf10, Creb3l2, Ywhae, **Pik3r1**, Ibsp, Ppp2r1b, Pi3kr3, Col2a1, Gsk3b, Efna5, Epor, Ywhah, Pdgfb, Bcl2l1, Kdr, Col3a1, Ddit4, Il7r, Map2kr1, Fgf7, Col6a6, Ppp2r2a, Thbs1, Col6a3, **Insr**, Reln, Igf1, Eif4b, Ngf, Ccne2, Creb1, Fgf11, Col1a1, Creb5, Col27a1, Fgfe1, Phlpp2, Itga5, **Irs1**, Ywwhab, Prlr, Itga11, Vegfa, Ghr, Col4a4, Itga10, Kras, Mapk3, Prkaa1, Ccne1, Nras, Rac1, Lamc3, Igf1r, Sos1, Bcl2, Col5a2, Col3a6, Ywhaq, Osmr, Col24r1, Col5a3, Pdgfb, Efna3, Sgk2, Chuk, Ppp2r3c, Gng5, Col1a2, Lamc1, Col4a1, Prkcz, **Ptb1b**, Fgf16, Fgf9, Col11a1, Bcl2l11, Ppp2r2c, Csf1, Itga7, Mcl1, Sgk1, Il6, Rps6kb2, **Pten**, Col6a2, Myb, Itgb8, Atf2, Col4a2, Col5a1, Fgf5, Pdgfa, Itga9 |
| FoxO signaling pathway | 0.0004 | 18 | Prkaa, Sos2, **Pi3kr1**, Pdpk1, Pik3r3, Ep300, **Irs4**, Fbxo32, Il7r, Map2k1, Atg12, **Insr**, Igf1, Plk3, **Irs1**, Tgfbr1, Usp7, Kras, Mapk3, Braf, Prkaa1, Mapk9, Il10, Homer2, Nras, Igf1r, Sos1, Sgk2, Prkab2, Chuk, Araf, Stk4, Prmt1, Bcl2l11, Prkag2, Gadd45a, Sgk1, Il6, Sirt1, S1pr1, **Pten**, Smad2, Homer1, Mapk8, Crebbp |
| MAPK signaling pathway | 0.0014 | 17 | Prkaa2, Sos2, **Pik3r1**, Pdpk1, Pik3r3, Ep300, **Irs4**, Fbxo32, Il7r, Map2k1, Atg12, **Insr**, Igf1, Plk2, **Irs1**, Tgfbr1, Usp7, Kras, Mapk3, Braf, Prkaa1, Mapk9, Il10, Homer2, Nras, Igf1r, Sos1, Sgk2, Prkab2, Chuk, Araf, Stk4, Prmt1, Bcl2l11, Prkag, Gadd45a, **Scl2a4**, Il6, Sirt1, S1pr1, **Pten**, Smad2, Homer1, Mapk8, Crebbp |
| AMPK signaling pathway | 0.0079 | 18 | Prkaa2, Tsc1, Ulk1, Creb3l2, **Pi3kr1**, Pp2r1b, Pdpk1, Pi3kr3, **Irs4**, Pp2r2a, **Insr**, Igf1, Rab8a, Creb1, Creb5, **Irs1**, Pfkfb2, Mlycd, Rab2a, Prkaa1, Igf1r, Ppargc1a, Adipor2, Prkab2, Ppp2r3c, Stradb, Rab14, Ppp2r2c, Prkag2, Pparg, Crtc2, Sirt1, Rps6kb2, **Scl2a4**, Cftr, Map3k7, Cab39 |
| Ras signaling pathway | 0.0079 | 17 | Sos2, Fgf1, Calm1, **Pik3r1**, Arf6, Pik3r3, Calm3, Efna5, Pdgfrb, Abl2, Bcl2l1, Pla2g2e, Kdr, Rap1a, Pla2g2f, Map2k1, Fgf7, **Insr**, Igf1, Ngf, Fgf11, Pak1, Fgf1, Nf1, Rassf5, Vegfa, Rala, Kras, Mapk3, Rab5b, Mapk9, Nras, Rac1, Igf1r, Sos1, Mras, Rasa1, Tiam1, Pdgfb, Efna2, Chuk, Gng5, Elk1, Fgf16, Stk4, Fgf9, Csf1, Prkacb, Rin1, Pak2, Fgf5, Pdgfa, Mapk8, Pak4 |
| cAMP signaling pathway | 0.0087 | 17 | Calm1, Creb3l2, **Pik3r1**, Gria3, Pik3r3, Ep300, Ppp1r1b, Adcy2, Calm3, Gria2, Slc9a1, Gnai3, Rap1a, Map2k1, Pde3a, Rock1, Atp1a1, Campk2b, Atp2b1, Tshr, Creb1, Pak1, Creb5, Adcyap1r1, Drd1a, Pde4a, Htr4, Mapk3, Braf, Mapk9, Atp2a, Rac1, Rock2, Camk4, Gabbr1, Tiam1, Adcy10, Grin3a, Adrb2, Gria1, Ppp1cb, Pde3b, Acox1, Adrb1, Oxtr, Cacna1, Prkacb, Vipr2, Cftr, Atp1b4, Hcar2, Mapk8, Crebbp |
| Endocytosis | 0.0087 | 17 | Smad6, Pip5k1, Arf5, Arf6, Cblb, Rab11, Psd3, Jdr, Cltc, Arap1, Rab22a, Erbb4, H2-M2, Smurf1, Adrb3, Vps37a, Tgfbr1, Asap1, Stam2, Smap1, Cxcr2, Agap1, Cyth3, Rabep1, Zfyve9, Ap2m1, Tonslm Erbb3, Igf1r, Cyth1, Sh3glb1, Pard3, Rab11, Iqsec2, Adrb2, Gbf1, Stam, Eps15, Prkcz, Adrb1, Pdccd6ip, Cav1, Hspa8, Smad2, Epn2, Arrb1, Rab11, Samd7, Wwp1, Tfrc, Cav2, Ldlrap, Dab2, Ehd4 |
| Insulin signaling pathway | 0.0445 | 17 | Prkaa2, Tsc1, Sos2, Calm1, **Pi3kr1**, Pdpk1, Pik3r3, Gsk3b, Calm3, **Irs4**, Cblb, Map2k1, Pde3a, **Insr**, Prkar2a, **Irs1**, **Scl2a4**, Kras, Mapk3, Braf, Prkaa1, Mapk9, Nras, Sos1, Ppargc1a, Prkab, Ppp1r3f, Ppp1r3c, Ppp1cb, Pde3b, Elk1, Prkcz, Araf, Prkag2, Prkacb, Rps6kb2, Mapk8 |
| cGMP-PKG signaling pathway | 0.0464 | 17 | Mef2d, Calm1, Creb3l2, Mef2c, **Pik3r1**, Pik2r3, Adcy2, Calm3, **Irs4**, Gnai, Map2k1, Ppp3r2, Pde3a, **Irs1**, Rock1, Atp1a1, Atp2b1, Creb1, Nfatc3, Creb5, Adrb3, **Insr**, Prkce, Mapk3, Atp2a, Gtf2i, Mylk3, Rock2, Slc8a2, Adrb2, Ppp1cb, Pde3b, Adrb1, Gucy1a2, Cacna1f, Itpr1, Mylk4, Atf2, Sfr, Kcnma1, Prkg1 |

**Figure S1. Effect of age and caloric restriction on insulin signaling in insulin-sensitive tissues.** 3m, 12m and 12mCR mice were injected with either saline (NaCl 0.9%) or insulin (10U/kg body weight) and after 10 minutes, tissues were collected. AKT phosphorylation was assessed by Western blotting in liver (A) and gastrocnemius muscle (B). The protein bands were quantified, and the fold change was calculated with respect to the control group (saline). Fold changes were compared between the experimental groups in each tissue and the percentage of change/recovery was stated. * p < 0.05, ** p < 0.01, *** p < 0.001, insulin *vs* saline; n = 4-6 animals/group.

**
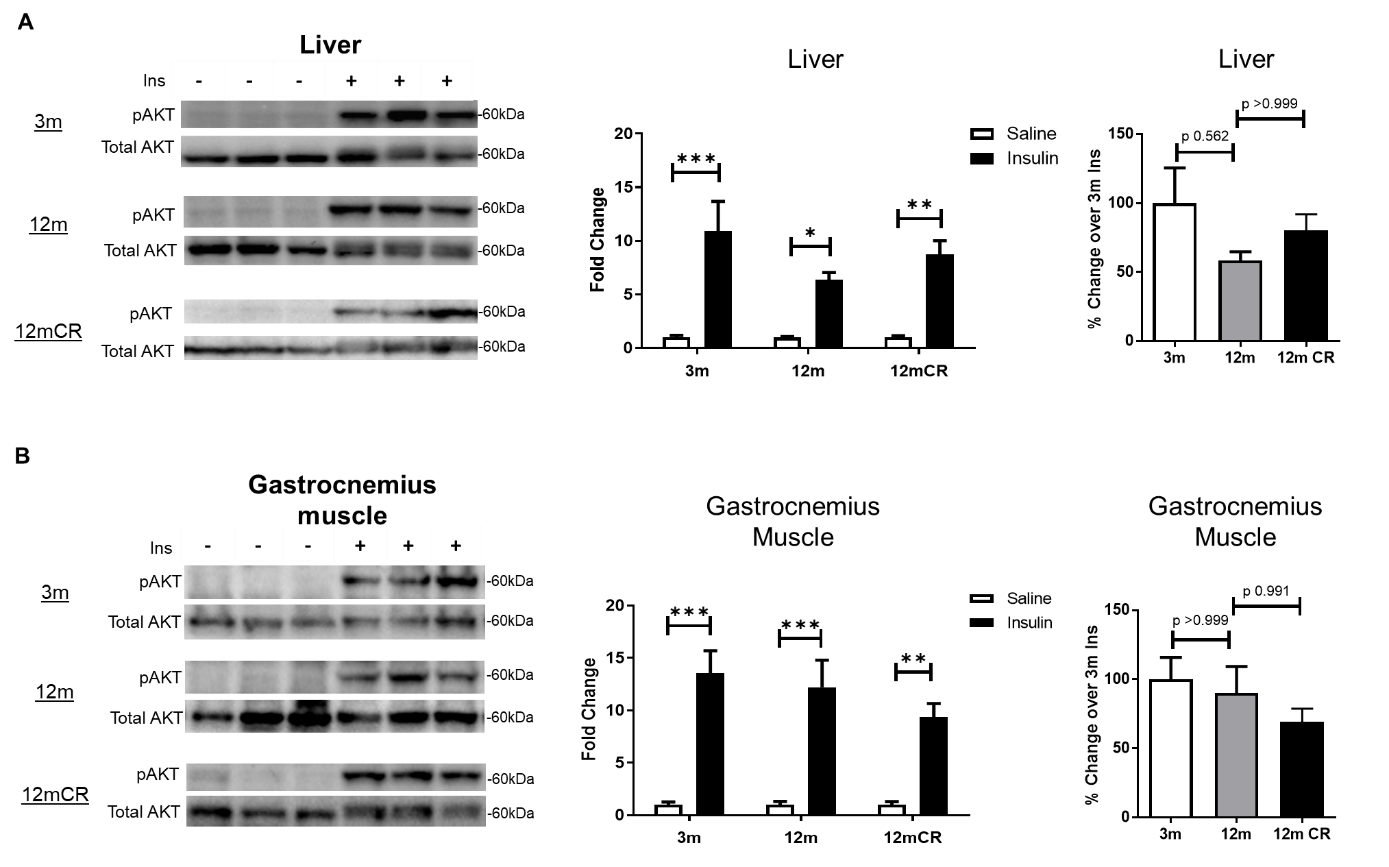
**

**Figure S2. Impact of age and caloric restriction on miRNAs in subcutaneous adipose tissue (continuation).** (A) Complete association network between matching miRNAs within 3m vs 12m and 12m vs 12mCR comparisons, including all putatively target genes involved in "Insulin signaling pathway", "Insulin resistance" and "PI3K-Akt signaling pathway" using KEGG pathway analysis. The validated target genes in the present work are highlighted in the network. miRNet was used for the representation. (B) *Slc2a4* mRNA expression correlating with physiological HOMA_IR_ in 3m, 12m and 12mCR mice. (C and D) *Insr* and *Dicer1* mRNA expression correlating with *Slc2a4* mRNA expression.


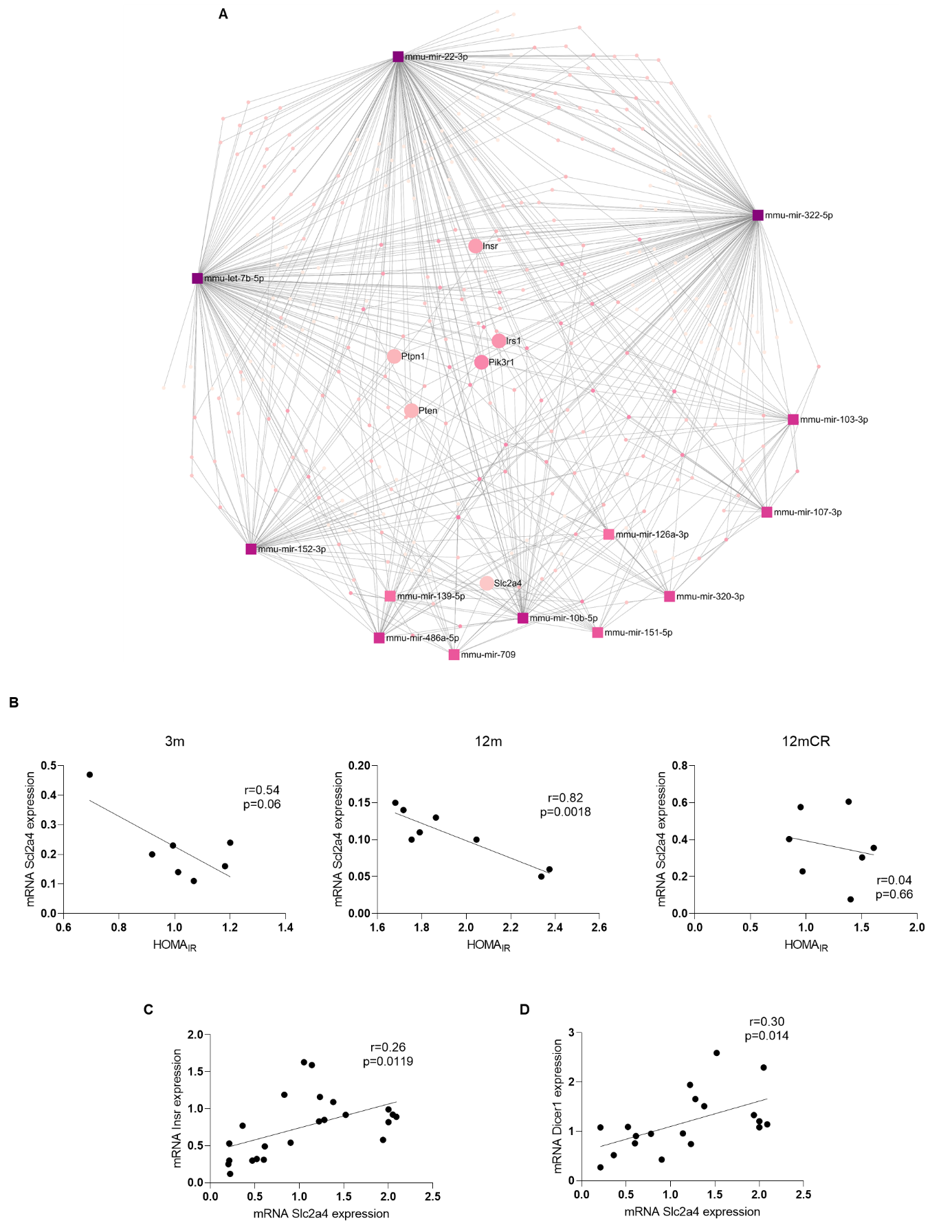


**Figure S3. Impact of age and caloric restriction on miRNAs in epidydimal adipose tissue.** (A) Heatmap and volcano plots representing the quantification of miRNAs expressed in the epidydimal white adipose tissue (eWAT) of mice at 3 and 12 months of age fed *ad libitum* (3m and 12m) and at 12 months of age after a long-term of caloric restriction (12mCR). (B) Venn diagram showing interactions between differentially expressed miRNAs (logFC ≥ 1 and logFC < -1; Adj. P-value < 0.05) in the comparisons 12m *vs* 3m and 12mCR *vs* 12m. (C) *Dicer1* mRNA quantification by qRT-PCR in eWAT (n = 7-8 animals/group). (D) and (E) Expression of mRNA levels of genes modulated by miRNAs -*Insr, Irs1, Pik3r1, Pten, Ptpn1 and Slc2a4*-,measured by qRT-PCR (n = 7-8 animals/group). (F) Cellular localization and trafficking of Glut4 glucose transporter in insulin-responsive eWAT, measured by immunofluorescence (magnification 1000x, scale bar = 20 µm). The arrowheads point to membrane-localized GLUT4. Graphs show the quantification of the fluorescence intensity of the cell membrane-localized GLUT-4 (* p < 0.05, ** p < 0.01, Insulin *vs* Saline).


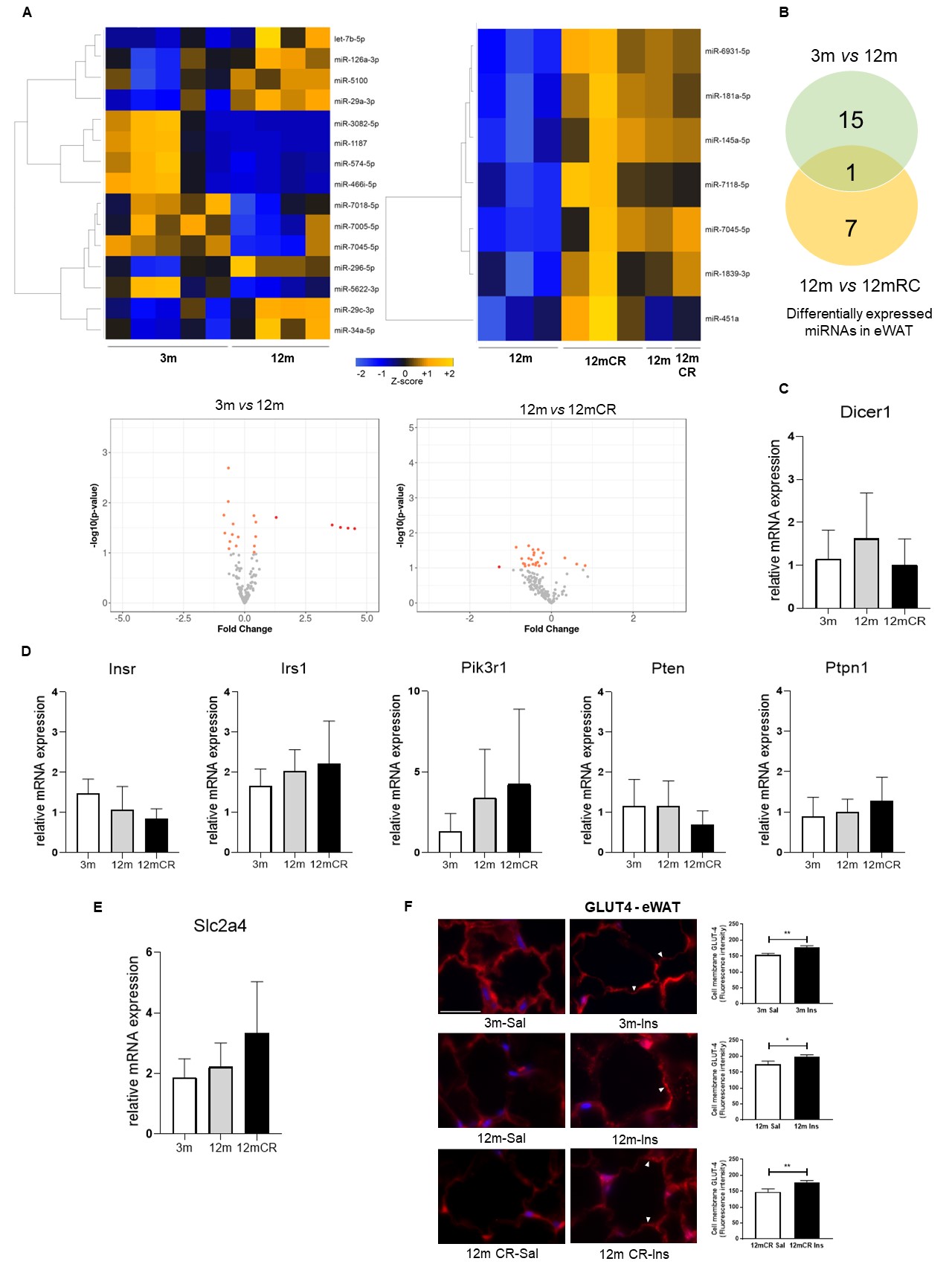

Supplement: Supplementary file 1 — Appendix S1. [file ACEL-22-e13919-s001.docx]
